# Insight Into Body Size Evolution in Aves: Based on Some Body Size‐Related Genes

**DOI:** 10.1111/1749-4877.12927

**Published:** 2024-12-11

**Authors:** Chaoyang Luo, Xionghui Xu, Chengfa Zhao, Qiuping Wang, Rongxing Wang, Datian Lang, Juan Zhang, Wenxian Hu, Yuan Mu

**Affiliations:** ^1^ Institute of Eastern‐Himalaya Biodiversity Research Dali University Dali Yunnan China; ^2^ Department of Agronomy and Life Science Zhaotong University Zhaotong Yunnan China; ^3^ Key Laboratory of Ecological Adaptive Evolution and Conservation on Animals‐Plants in Southwest Mountain Ecosystem of Yunnan Province Higher Institutes College, School of Life Sciences Yunnan Normal University Kunming Yunnan China; ^4^ Erhai Watershed Ecological Environment Quality Testing Engineering Research Center of Yunnan Provincial Universities, Erhai Research Institute West Yunnan University of Applied Sciences Dali Yunnan China; ^5^ Collaborative Innovation Center for Biodiversity and Conservation in the Three Parallel Rivers Region of China Dali Yunnan China

**Keywords:** body size, convergent/parallel evolution, correlation, positive selection

## Abstract

Birds exhibit remarkable variations in body size, making them an ideal group for the study of adaptive evolution. However, the genetic mechanisms underlying body size evolution in avian species remain inadequately understood. This study investigates the evolutionary patterns of avian body size by analyzing 15 body‐size‐related genes, including *GHSR*, *IGF2BP1*, and *IGFBP7* from the growth hormone/insulin‐like growth factor axis, *EIF2AK3*, *GALNS*, *NCAPG*, *PLOD1*, and *PLAG1* associated with tall stature, and *ACAN*, *OBSL1*, and *GRB10* associated with short stature, four genes previously reported in avian species: *ATP11A*, *PLXDC2*, *TNS3*, and *TUBGCP3*. The results indicate significant adaptive evolution of body size‐related genes across different avian lineages. Notably, in the *IGF2BP1* gene, a significant positive correlation was observed between the evolutionary rate and body size, suggesting that larger bird species exhibit higher evolutionary rates of the *IGF2BP1* gene. Furthermore, the *IGFBP7* and *PLXDC2* genes demonstrated accelerated evolution in large‐ and medium‐sized birds, respectively, indicating distinct evolutionary patterns for these genes among birds of different sizes. The branch‐site model analysis identified numerous positively selected sites, primarily concentrated near functional domains, thereby reinforcing the critical role of these genes in body size evolution. Interestingly, extensive convergent evolution was detected in lineages with larger body sizes. This study elucidates the genetic basis of avian body size evolution for the first time, identifying adaptive evolutionary patterns of body size‐related genes across birds of varying sizes and documenting patterns of convergent evolution. These findings provide essential genetic data and novel insights into the adaptive evolution of body size in birds.

## Graphical abstract

These findings provide new insights into the genetic determinants of body size evolutionary mechanisms in Aves.

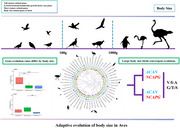

## 1 Introduction

Body size is acknowledged as a prominent and significant phenotypic trait in animals, characterized as a typical quantitative and complex trait that exhibits continuous variation (Hensen et al. 2004; Sibly and Brown 2007; Kemper, Visscher, and Goddard 2012). In the field of evolutionary biology, body size represents a compelling subject that has consistently garnered attention (Ozgul et al. 2010). Investigating the evolutionary mechanisms associated with body size is essential for a comprehensive understanding of phenotypic adaptability and for elucidating the regulatory mechanisms that underlie the evolution of body size.

Birds constitute an exceptionally diverse clade within the animal kingdom, encompassing over 10 000 extant species worldwide (Tobias et al. 2022). Owing to their varied life histories and adaptations to different habitats, birds exhibit considerable variation in body size (Meiri and Dayan 2003; Yom‐Tov and Geffen 2006; Guillemain et al. 2010; Ryding et al. 2021), with an extraordinary 41 000‐fold difference between the smallest and largest species (Maurer 1998). For instance, the weight of the hummingbird (*Calypte anna*) is approximately 2 g, while the ostrich (*Struthio camelus*) can exceed 100 kg. Notably, despite this vast disparity, more than half of bird species weigh less than 38 g (Tobias et al. 2022). Blackburn and Gaston (1994) conducted an analysis of the weight data for two‐thirds of currently recognized bird species, documenting the frequency distribution of their weights and observing that even after log‐transforming the weight axis, the distribution remains significantly right‐skewed. This finding underscores that the smallest body size categories do not correspond to the highest species diversity. Furthermore, substantial body size variations are evident within certain bird orders. For example, within the Galliformes, the smallest species, *Synoicus adansonii*, weighs approximately 40 g, whereas the largest species, *Meleagris gallopavo*, weigh up to 5700 g, resulting in a nearly 140‐fold difference (Tobias et al. 2022). Consequently, birds are considered an ideal group for investigating the evolutionary mechanisms underlying animal body size due to their extensive range of body sizes.

The evolution of avian body size is a multifaceted process influenced by a variety of factors. A comprehensive understanding of these factors is essential for elucidating the mechanisms underlying bird evolution. One significant factor affecting bird body mass is the ecological environment. The availability of food resources directly impacts the weight and size of birds. Jetz et al. (2012) demonstrated that the global diversity of avian species is closely linked to the spatial and temporal distribution of food resources. Additionally, habitat characteristics exert a considerable influence on bird body size. For instance, birds inhabiting dense forests tend to be smaller, which facilitates maneuverability in complex environments, whereas birds residing in open areas may exhibit larger body sizes to enhance their capacity for long‐distance flight. Furthermore, habitat diversity and the availability of food resources also significantly affect the evolution of avian body size, particularly under extreme climatic conditions. According to Bergmann's rule, birds inhabiting colder climates are generally larger, which serves to minimize heat loss (Meiri and Dayan 2003). Fan et al. (2019) confirmed this rule, finding significant variations in bird body mass across different climatic conditions. Temperature and precipitation are likely primary factors driving long‐term evolutionary trends in avian body mass (Yom‐Tov and Geffen 2006). Meanwhile, it is widely accepted that the metabolic rate is inversely correlated with body mass (Calder 1974; McNab 2009). In recent years, advancements in genomic availability and the proliferation of genome‐wide association studies (GWAS) have revealed that mutations in specific genes associated with poultry breeding can significantly enhance body mass (Zhou et al. 2018; Deng et al. 2019). This suggests that the evolution of relevant genes and pathways has played a critical role in the evolution of body mass. However, the molecular genetic mechanisms underlying the evolution of bird body size remain largely unexplored.

Research on body size evolution across various animal groups has identified several key genes and their roles in the regulation of body size. For instance, in mammals, variations in the *NCAPG* (Non‐SMC Condensin I Complex Subunit G) and *PLAG1* (Pleomorphic Adenoma Gene 1) genes are critical roles during embryonic growth phases, which are closely linked to body size regulation, particularly demonstrating significant effects in cattle (Takasuga 2016; La et al. 2019). In cetaceans, genes associated with body size exhibit positive selection pressure and distinct evolutionary patterns (Sun et al. 2019; Silva et al. 2023). In carnivores, genes related to increased body size are subject to selection and are primarily associated with cancer resistance, thereby enabling larger carnivores to mitigate cancer risk (Huang et al. 2021). In squamates, particularly among snakes, genes associated with body size show elevated evolutionary rates (Wu et al. 2022). These findings suggested that the evolution of body size is intricately linked to the selection of relevant genes and pathways.

This study builds upon previous research concerning the evolution of body size by selecting 15 genes that are closely associated with this trait (e.g., *GHSR*, *EIF2AK3*, *ACAN*, etc.) to address the following three scientific questions: (1) Have the genes related to body size undergone adaptive evolution throughout avian evolution? Do these genes display distinct evolutionary patterns across bird lineages characterized by varying body sizes? (2) Is there a correlation between body size‐related genes and morphological variables associated with avian body sizes? (3) Have these body size‐experienced genes undergone convergent evolution? The objective of this research is to elucidate the genetic underpinnings of body size evolution in birds, thereby providing new insights into the evolution of body size in animals.

## 2 Materials and Methods

### 2.1 Collection and Division of the Data

Body mass is widely recognized as a primary and effective metric for examining the size, behavior, and evolutionary history of animal species (Herberstein et al. 2022; Do et al. 2023). According to previous studies (Tiersch and Wachtel 1991; Gaston and Blackburn 1995; Saracco et al. 2005; Field et al. 2013; Zimova et al. 2023), we have preliminarily classified birds into three main size categories based on body mass: large (body mass ≧ 1001 g), medium (101 g < body mass ≦ 1000 g), and small (body mass ≦ 100 g). Utilizing the classification, the present study employed a stratified sampling method to select 56 bird species of varying sizes from these defined body mass ranges, encompassing 20 orders and 36 families (dataset 1). The body mass data for the selected avian species were sourced from Tobias et al. (2022), which adequately accounted for the effects of sexual dimorphism and provided average values. Among the selected species, 19 were classified as large‐bodied, representing 33.93% of the sample; 20 were classified as medium‐bodied, accounting for 35.71%; and 17% were classified as small‐bodied, comprising 30.36%. Furthermore, we subdivided body mass into three levels (small, medium, and large) within each of the three categories, selecting four to five species for each level to ensure a relatively continuous distribution of body mass across the overall sample. This meticulous selection process aims to capture the extensive diversity of avian body sizes as comprehensively as possible, thereby enhancing the accuracy and reliability of the results (Table S1a). To further investigate the evolutionary patterns of closely related species that may exhibit variations in body size, we constructed two additional sub‐datasets at the order level: Galliformes (10 species, dataset 2, Table S1b) and Sphenisciformes (11 species, dataset 3, Table S1c), and conducted analogous analyses.

### 2.2 Selection of Candidate Genes

The regulation of body size is governed by a complex interplay of critical genes and biological pathways. Drawing upon previous research (Zhou et al. 2018; Deng et al. 2019; Sun et al. 2019; Wu et al. 2022; Silva et al. 2023), a total of 15 candidate genes have been identified and categorized into four distinct groups for subsequent analysis (Table S2). These groups include three genes (*GHSR*, *IGF2BP1*, and *IGFBP7*) that are part of the growth hormone/insulin‐like growth factor axis, seven genes associated with tall stature (*EIF2AK3*, *GALNS*, *NCAPG*, *PLOD1*, and *PLAG1*), three genes linked to short stature (*ACAN*, *OBSL1*, and *GRB10*), and four genes implicated in body size variation in avian species (*ATP11A*, *PLXDC2*, *TNS3*, and *TUBGCP3*). The coding DNA sequences (CDS) for each gene were retrieved from the National Center corresponding to Biotechnology Information (NCBI) database (https://www.ncbi.nlm.nih.gov/). The accession numbers for each gene are detailed in Table S3.

### 2.3 Processing of the Sequences

The CDS for each gene were aligned utilizing the webPRANK tool (https://www.ebi.ac.uk/goldman‐srv/webprank/), which employs a codon methodology approach (Löytynoja and Goldman 2010), manually refined in MEGA 7 (Kumar et al. 2018). Given that the estimation of evolutionary parameters, the quality of sequence alignments should be considered. Thus, the coverage should be more than the half when blastn to the genome, respectively, as well as some degenerate bases have been removed to minimize the rate of false‐positive predictions. Only high‐quality sequences were retained for subsequent analyses.

### 2.4 Selection Pressure Analysis

Natural selection is a process through which organisms that possess advantageous adaptations to their environment are more likely to survive and reproduce, whereas those that are less well‐adapted face a higher risk of elimination. The comparison of nonsynonymous (*d*_N_) to synonymous (*d*_S_) substitution ratios has emerged as a valuable method for quantifying the effects of natural selection on molecular evolution (Kimura 1983; Yang et al. 2000); namely, the ω value, serves as an indicator of the molecular evolutionary rate. Specifically, values of ω < 1, = 1, and > 1 are indicative of purifying selection, neutral evolution, and positive selection, respectively.

To estimate the ω values, we utilized the codon‐based maximum likelihood models as implemented in the CodeML program of PAML 4.9 (Yang 2007), using the likelihood ratio test (LRT) was employed to evaluate significant differences between the two models by calculating the chi‐square (χ^2^) distribution of the statistic (2ΔL) in relation to the degrees of freedom. Indicators of positive selection were identified through Bayesian Empirical Bayes (BEB) analysis, with posterior probabilities (PP) of ≥ 0.8 considered significant (Yang et al. 2005). For subsequent analyses, we accessed the TimeTree database (http://www.timetree.org/) and BirdTree (http://birdtree.org/) to obtain phylogenetic data, integrating information from prior studies (Prum et al. 2015). These methodologies collectively enhance the accuracy of the analysis, thereby facilitating a more precise understanding and interpretation of the role of natural selection in the adaptive evolution of species.

### 2.5 Branch Model Analyses

To investigate the evolutionary rate across the entire avian lineage, we utilized the one‐ratio (M0) model implemented in the CodeML program of the PMAL 4.9 package (Yang 2007). The M0 model posits that all species within the dataset are subjected to the same selective pressure, which implies a uniform ω value across all branches and nodes of the phylogenetic tree. Subsequently, we compared the M0 model with the free ratio model (M1) to determine whether evolutionary lineages exhibit varying evolutionary rates. The free ratio model allows for the assumption that each branch possesses an independent ω value, which was also implemented via the CodeML program in PAML 4.9 (Yang 2007). To further investigate whether there are significant differences in evolutionary rates among avian lineages of varying body sizes, we extracted ω values from each terminal branch (N × *d*_N_ and S × *d*_S_ equal to 0 are excluded). Rigorous statistical tests were conducted, including normality testing and the removal of outliers. Subsequently, a one‐way analysis of variance (ANOVA) was performed using IBM SPSS 20.0 (Jinn 2011). The significance level is set at *p* < 0.05.

### 2.6 Branch‐Site Model Analyses

To determine whether sites subject to positive selection are restricted to specific evolutionary lineages, the more rigorous branch‐site model was employed using the CodeML program from PAML 4.9 (Yang 2007). This model permits each site on a designated branch to possess its own ω value, initially categorizing the phylogeny into foreground branches (those of primary interest) and background branches. The alternative hypothesis model Ma (positive selection model: 0 < ω_0_ < 1, ω_1_ > 1, and ω_2_ ≥ 1) is compared to the null hypothesis model Ma0 (neutral model: 0 < ω_0_ < 1, ω_1_ = 1, and ω_2_ = 1). In this investigation, each terminal branch was treated as a foreground branch. When the pairwise comparison of models (Ma vs. Ma0) shows significant differences and ω > 1, the positive selection model Ma is accepted. Additionally, sites with a posterior probability (PP) > 0.8 are considered to be under positive selection. The significance of all pairwise models was determined through LRTs based on chi‐square tests, with a significant level at *p* < 0.05 (Anisimova and Yang 2007).

### 2.7 Association Analysis Between Gene Evolution and Phenotypes

To investigate the potential relationships between gene evolutionary rates and body size phenotypes, we employed the methodology outlined by Montgomery, Mundy, and Barton (2014), calculating root‐to‐tip ω values for each avian species. These calculations involved determining the average ω value from the last common ancestor (LCA) to each extant terminal species. Root‐to‐tip ω values provide a more comprehensive reflection of a gene evolutionary trajectory, making them more suitable for regression analyses with phenotypic data from contemporary species within the Aves. Furthermore, if an individual ω value was identified as excessively high or low during statistical testing, we designated such ω values as “n/a” to mitigate the influence of outliers on the overall root‐to‐tip ω analysis. To enhance the normality for subsequent regression analyses, all root‐to‐tip ω values were transformed to their log10 equivalents (Montgomery, Mundy, and Barton 2014). The branch model (free ratio) was employed to estimate the average ω from the ancestral bird to each terminal species. The phenotypic traits, including body mass of representative avian species, were sourced from previously published data (Tobias et al. 2022; Table S2). Phylogenetic generalized least squares (PGLS) regression was utilized to analyze the relationship between log (root‐to‐tip ω) and log (body mass). The lambda (λ) value, estimated using the maximum likelihood method, served as a quantitative measure of phylogenetic signals (Pagel 1999). All statistical analyses were conducted using R version 4.2.3, employing the Caper package (Kembel et al. 2010; R Core Team 2023).

### 2.8 Parallel/Convergent Evolution Analysis

To investigate whether these genes exhibit convergent adaptations in large avian lineages across distinct phylogenetic frameworks, we conducted a comprehensive analysis of parallel and convergent evolutionary changes. Initially, we reconstructed the ancestral amino acid sequences for each dataset utilizing the M0 model approach, as implemented in the CodeML program within the PAML package (Yang 2007). We subsequently identified parallel and convergent amino acid substitution sites among branches representing convergent large‐bodied bird lineages (branches a–l, Figure S1). Following this, we employed CONVERGE 2 to assess whether the observed parallel and convergent substitutions in the focal branches were fixed randomly or through the process of natural selection (Zhang and Kumar 1997). A statistical test was performed to compare the observed number of parallel and convergent amino acid substitutions against the expected value. A significance level of *p* < 0.05 indicates that observed substitutions were influenced by natural selection rather than occurring due to random mutation.

### 2.9 Structural and Functional Analysis

To enhance our understanding of the functional significance of the positively selected sites identified through the branch‐site model, as well as the parallel and convergent amino acid sites, we conducted a comprehensive search for information regarding functional sites and domains using the UniProt website (http://www.uniprot.org/) (UniProt Consortium 2019) and InterPro (https://www.ebi.ac.uk/interpro/) (Paysan‐Lafosse et al. 2023).

## 3 Results

### 3.1 The Results of M0 and M1 Model

The results from model M0 indicated that 15 body‐size‐related genes exhibited ω values ranging from 0.029 for *PLAG1* to 0.343 for *NCAPG* in dataset 1 (Table S4a), from 0.025 for *PLAG1* to 0.370 for *TNS3* in dataset 2 (Table S4b), and from 0.0001 for *PLAG1* to 0.551 for *GRB10* in dataset 3 (Table S4c). These values are significantly below 1, suggesting that these genes are subject to strong purifying selection, which constrains their functional roles in the development of avian body size. Further analysis utilizing the M1 model revealed significant differences in evolutionary rates among 11 of these genes in dataset 1 (*ACAN*, *EIF2AK3*, *IGFBP7*, *NCAPG*, *OBSL1*, *ATP11A*, *PLXDC2*, *TNS3*, *TUBGCP3*, *PLAG1*, and *GALNS*; Table S5a), 11 genes in dataset 2 (*EIF2AK3*, *GALNS*, *IGFBP7*, *NCAPG*, *OBSL1*, *GRB10*, *IGF2BP1*, *ATP11A*, *PLXDC2*, *TNS3*, and *TUBGCP3*; Table S5b), and 3 genes (*GHSR*, *GRB10*, *OBSL1*) in dataset 3 (Table S5c). These findings indicate that, despite the prevalent purifying selection pressure, significant various in evolutionary rates persist among clades across different orders.

### 3.2 The Evolutionary Rates Among Different Body Size Lineages

**FIGURE 1:**
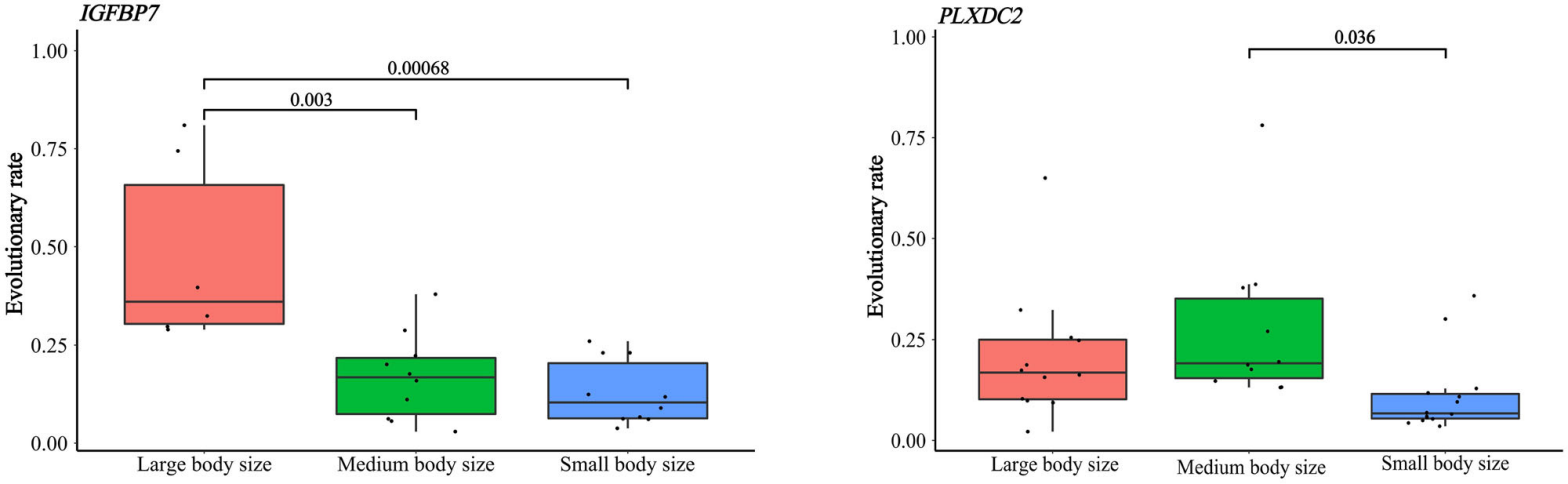
Evolutionary rates about *IGFBP7* and *PLXDC2* gene among different body size birds.

To further evaluate the evolutionary rates of extant species across various body size lineages, the ω values derived from the free‐ratio model for each terminal branch were extracted, and one‐way ANOVA was conducted using SPSS. Notably, for the *IGFBP7* gene, the evolutionary rates of large‐bodied birds were significantly higher than those of medium‐bodied and small‐bodied birds in dataset 1 (Figure 1). Additionally, the evolutionary rates of the *PLXDC2* gene were relatively higher in medium‐bodied birds, and significantly greater than those observed in small‐bodied birds (Figure 1).

### 3.3 The Results of Branch‐Site Model Among Terminal Lineages

**FIGURE 2:**
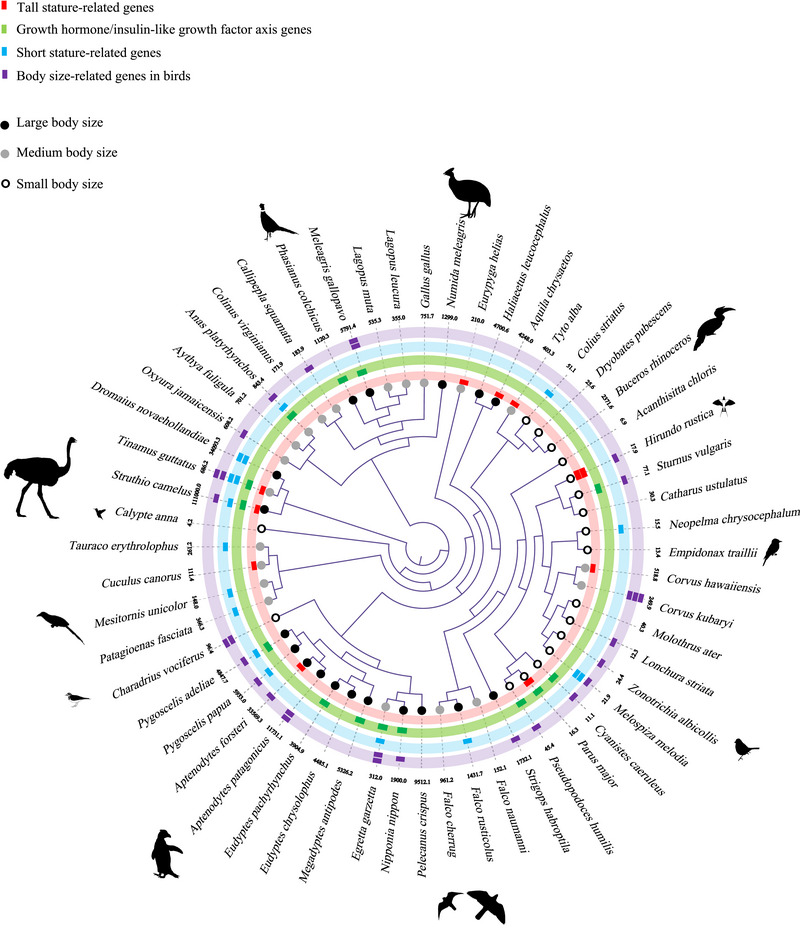
The distribution of positive selection detected by the branch‐site model across avian lineages. Red, green, blue, and purple rectangles represent the position of positive selection signals for genes associated with tall stature, growth hormone/insulin‐like growth factor axis, short stature, and body size in birds, respectively. The black, gray, and empty circles at the end of each terminal branch represents large, medium, and small body sizes for birds, respectively. And the numbers nearby the species name are the body mass of each species [units: g].

The more stringent branch‐site model revealed that positive selected sites were detected among certain terminal branches in 13 genes from dataset 1, including *ACAN*, *EIF2AK3*, *IGFBP7*, *IGF2BP1*, *NCAPG*, *OBSL1*, *GALNS*, *PLAG1*, *PLOD1*, *ATP11A*, *PLXDC2*, *TNS3*, and *TUBGCP3*. In total, 784 sites were identified to be under positive selection distributed across 30 terminal branches (Figure 2; Table S6). Among these, *ACAN*, *OBSL1*, *IGF2BP1*, *TUBGCP3*, *TNS3*, and *PLXDC2* were detected positive selection among large‐, medium‐, and small‐bodied groups, in which 725 sites were identified, accounting for 92.48% of the total; *IGFBP7*, *NCAPG*, *GALNS*, and *ATP11A* were detected positive selection among large‐ and medium‐bodied groups, in which 52 sites were identified, accounting for 6.63% of the total; *GRB10*, and *EIF2AK3* were detected positive selection among medium‐ and small‐bodied groups, in which 5 sites were identified, accounting for 0.63% of the total; only two branches including sites were detected under the positive selection, *PLAG1* and *PLOD1*; however, the positive selection merely focused on the small‐bodied group, in which 2 sites were identified, accounting for 0.26% of the total (Figure 2; Table S6). We further analyzed the positively selected sites and found that, although the same positively selected genes were present among different body size groups, the specific sites differed. Particularly, in both three body mass categories, *IGF2BP1* gene exhibited the highest number of positively selected sites, totaling 357, which accounts for 45.54% of the overall total. Particularly in the case of the extreme body mass ostrich (*Struthio camelus*), the *IGF2BP1* gene showed significant positive selection, with 97 positively selected sites, accounting for 12.37% of the total (Table S6).

**FIGURE 3:**
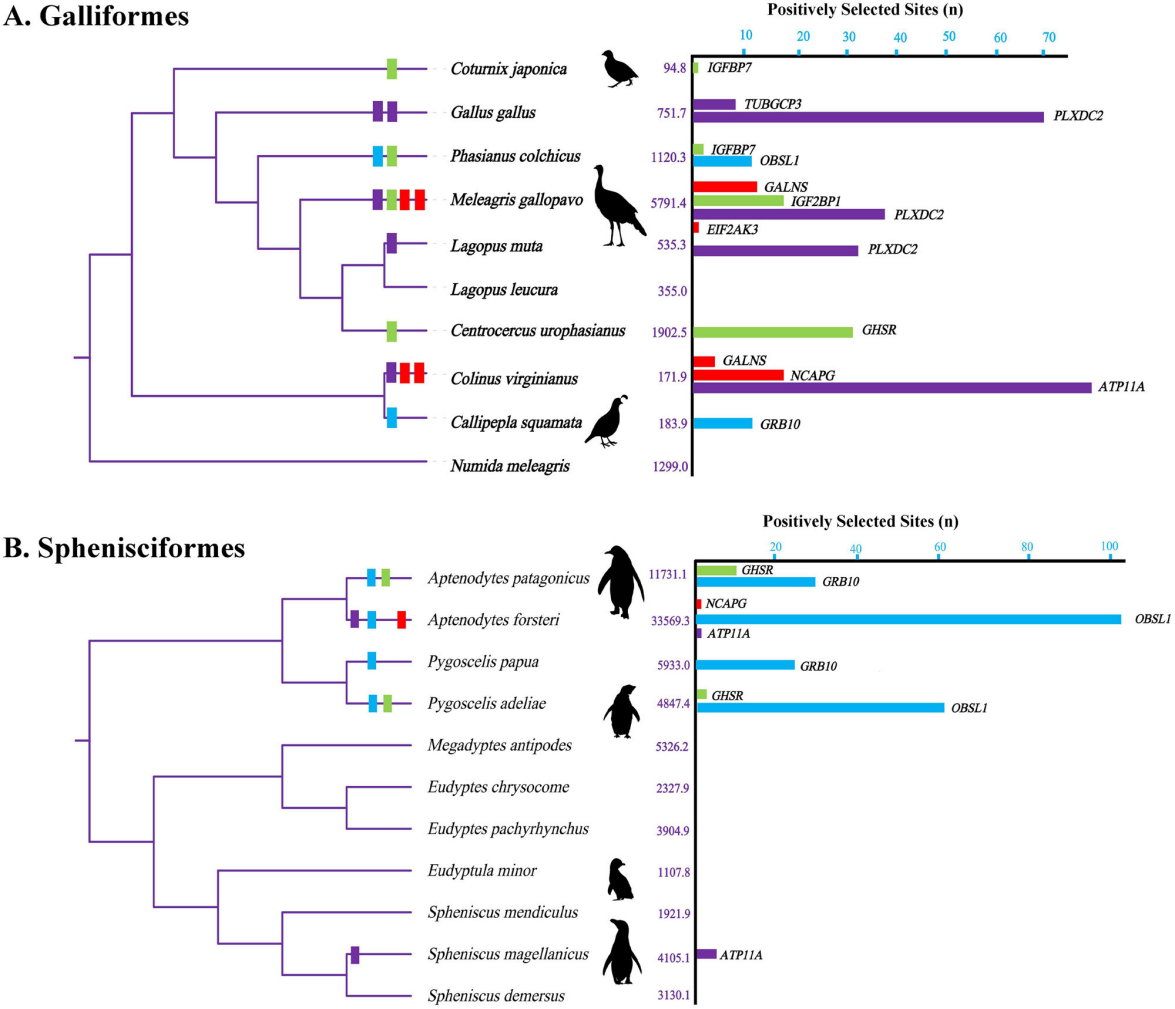
The statistics for positively selected signal distribution and number, A is Galliformes and B is Sphenisciformes.

At the same time, positively selected signals were detected in 6 out of 10 branches involving 321 sites in Galliformes (dataset 2). Among them, 37 positively selected sites were identified in tall stature‐related genes *NCAPG*, *EIF2AK3*, and *GALNS*; 46 positively selected sites were identified in *GHSR*, *IGFBP7*, and *IGF2BP1*; as well as 22 positively selected sites were identified in short stature‐related genes *GRB10* and *OBSL1*. Obviously, most of the positively selected signals mainly focus on *ATP11A*, *PLXDC2*, and *TUBGCP3* (Figure 3A; Table S6b), which were confirmed to be associated with body mass in poultry (Zhou et al. 2018; Deng et al. 2019). Besides, positively selected signals were detected in 5 out of 11 branches involving 172 sites in Sphenisciformes (dataset 3), while the positively selected signals mainly concentrate on short stature‐related genes *GRB10* and *OBSL1* (Figure 3B; Table S6c).

Further functional site annotation revealed that there are many positively selected sites located on or near important functional domains or functional sites within the three‐dimensional (3D) structure of the proteins, such as site 1122 in OBSL1, site 72 in PLAG1, sites 265–268 in ATP11A, site 392 in GALNS, and sites 176–178 in GRB10 (Table S7a–c).

### 3.4 Convergent Evolution Among Avian Lineages With Large Body Mass

In the analysis of 15 genes across various avian lineages (branch a—branch l in dataset 1, Figure S1), a total of 191 parallel amino acid substitution sites and five convergent amino acid substitution sites were identified (*p* < 0.05, Table S8). Convergent evolutionary signal sites were extensively observed across different lineages in 13 genes, specifically: *OBSL1*, *NCAPG*, *TNS3*, *ACAN*, *PLOD1*, *GHSR*, *EIF2AK3*, *PLXDC2*, *TUBGCP3*, *GALNS*, *IGFBP7*, and *IGF2BP1*. Notably, the focus of convergent evolution was primarily on the genes *ACAN*, *NCAPG*, and *OBSL1*, which collectively accounted for 145 sites, representing 74% of the total identified. In detail, the *ACAN* gene exhibited 53 parallel substitution sites and 3 convergent substitution sites, constituting 28.5% of the total. The *NCAPG* gene displayed 38 parallel substitution sites, accounting for 19% of the total, with two convergent substitution sites detected in the comparisons of branches f versus j and h versus l. The *OBSL1* gene contained 51 parallel substitution sites identified across various comparisons, including a versus d, e, g, h, j, k, l, d versus e, g, i, j, k, l, as well as e versus g, i, k, l, which represented 26% of the total. Additional genes included *TNS3*, which had 14 parallel substitution sites primarily the comparisons of a versus e, f, g, k, and d versus e, f, h; *EIF2AK3* with nine sites; *PLOD1* with seven parallel substitution sites; *TUBGCP3* with six sites; *GALNS* with four sites; *PLXDC2* and *IGFBP7* each with three parallel substitution sites; *IGF2BP1* with three parallel substitution sites located in the comparisons of a versus d, e versus i, and h versus j; and *GHSR* with one site (Table 1; Figure S1; Table S9).

**TABLE 1:** The partial results of parallel/convergent amino acid substitution sites.

Branches	Genes	Sites	AA change	Observed number	Expected number	*p* value
a vs. h	*GALNS* *OBSL1* *ACAN*	428 1023 1029 1430 1436	R‐Q A‐T R‐A I‐S V/I‐A	5	0	0
c vs. i	*ACAN*	328	V/A‐I	1	0	0
e vs. l	*TUBGCP3* *EIF2AK3* *OBSL1* *ACAN*	769 806 685 1702 1537 1764	R‐G H‐Q T‐M G‐E H/Q‐R I‐V	6	0	0
f vs. j	*NCAPG*	878	N/D‐E	1	0	0
h vs. l	*OBSL1* *NCAPG* *ACAN*	1065 683 1411	I‐V G/T‐S I‐T	3	0	0

Further functional site annotation has indicated that numerous substitution sites are located within critical functional domains. Specifically, sites 383, 734, and 328 in the ACAN are in proximity to glycosylation regions; sites 18, 79, 56, 848, 382, and 304 in the OBSL1 are situated within the region responsible for disulfide bond formation, while sites 258, 288, and 264 are located within the immunoglobulin domain. Additionally, sites 928 and 952 in the NCAPG are found in the modified residue region, and sites 240, 107, and 300 are located within the Armadillo‐type fold domain. Furthermore, site 342 in the GHSR is positioned within the topological domain (Table S9). Overall, genes associated with body size in large‐bodied bird lineages exhibit extensive convergent and parallel substitution events.

### 3.5 Association Between Gene Evolution and Body Mass

**FIGURE 4:**
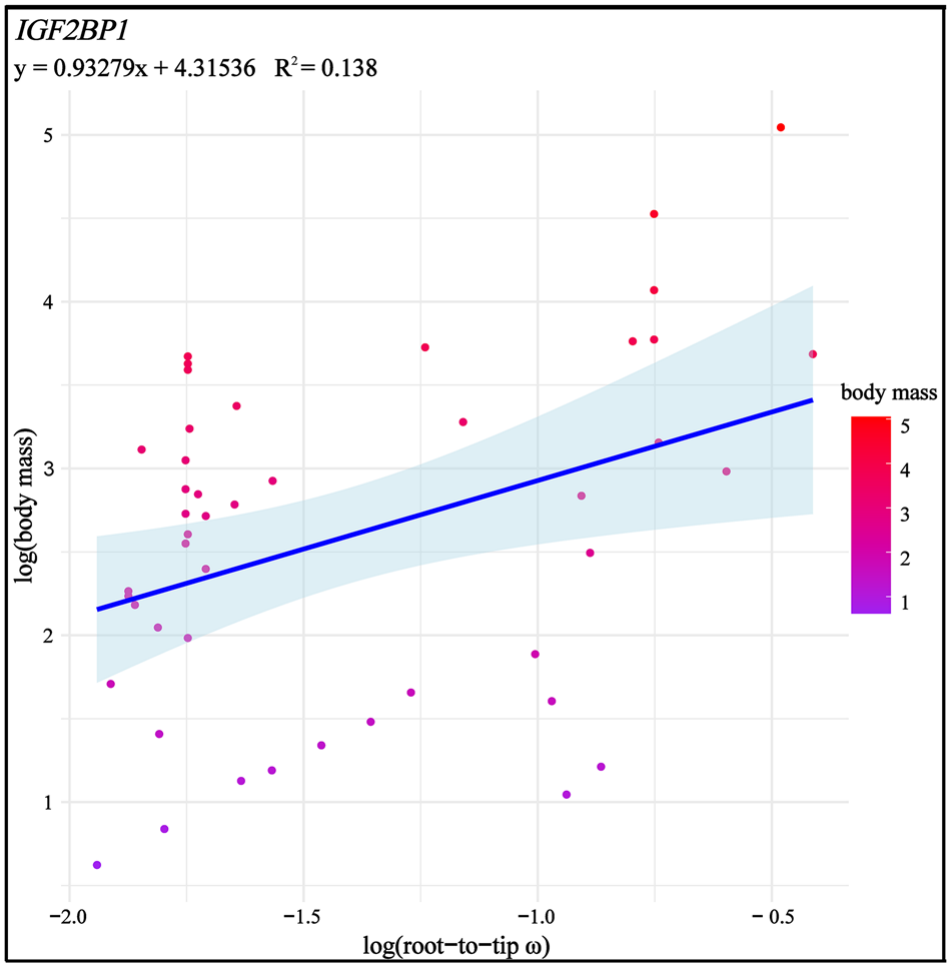
Correlation between evolutionary rates and body mass in *IGF2BP1*.

PGLS regression analyses indicated a significant positive association between the log (root‐to‐tip ω) and the log (body mass) at the *IGF2BP1* gene in dataset 1 (*R* ^2^ = 0.138, *p* = 0.0014, lambda = 1.000; Figure 4; Table S10). In contrast, no such association was observed for the remaining 14 genes in dataset 1, nor for any genes in datasets 2 and 3.

## 4 Discussion

The morphological evolution of body size in animals constitutes the fundamental framework for understanding diverse phenotypic adaptions (Ozgul et al. 2010). It is well established that extant birds exhibit a significant degree of diversity in body size. During the process of adaptive radiation, both large and small body sizes present distinct adaptive advantages and disadvantages (Tobias et al. 2022). In this study, we employed comparative genomics and bioinformatics methodologies to conduct a comprehensive analysis of body size‐related genes in avian species. Our findings reveal varying evolutionary patterns of body size‐related genes across different avian species characterized by distinct body sizes.

### 4.1 Divergent Selection in Different Body Size Lineages Among Related Genes

The body size–frequency distributions of extant avian species have primarily been focused on macroecological analyses (Clauset and Erwin 2008; Glazier 2008; Clauset, Schwab, and Redner 2009; Olson et al. 2009). However, the evolution of avian body size is a complex process influenced by multiple factors, including genetic components. Body mass is a multifaceted continuous trait that is regulated by different genes, gene families, and pathways. Notably, different genes may experience distinct selective pressures, as exemplified by those involved in tooth development (Machado et al. 2016). In this study, 15 genes were identified as having undergone divergent selective pressures (Table S4). Further analysis revealed that various lineages within avian phylogeny have also experienced divergent selective pressures (Table S5), suggesting that the evolutionary patterns of body size evolution experience divergence within the clade Aves. Additionally, accelerated evolutionary rates were observed in *IGFBP7* within the large‐bodied group and *PLXDC2* in the medium‐bodied group, respectively (Figure 1). Previous studies have found that *IGFBP7* is associated with body size increase and cancer suppression in giant whales (Silva et al. 2023). In this study, it is also shown to undergo accelerated evolution in large birds. As an upstream growth axis gene, this finding suggests a similarity in the evolutionary mechanisms shared by aquatic mammals and birds, underscoring the significance of *IGFBP7* as a key gene in the evolution of body mass.

Further analysis indicates that genes such as *ACAN*, *OBSL1*, *IGF2BP1*, *TUBGCP3*, *TNS3*, and *PLXDC2* are extensively distributed across various avian lineages and exhibit multiple signals of positive selection, highlighting their essential roles in the evolution of body size in birds. Notably, *TUBGCP3*, *TNS3*, and *PLXDC2* are significantly involved in the regulation of avian body size traits, as corroborated by prior research on poultry breeding (Zhou et al. 2018), suggesting that these genes may facilitate growth in body size among avian species. In contrast, *ACAN* and *OBSL1* are linked to dwarfism, with positive selection signals identified in these genes across all bird‐size categories. Given that mutations in the *ACAN* gene result in dwarfism phenotypes in mice, humans, and cattle (Li and Olsen 1997), we speculate that positive selection in the *ACAN* gene among smaller bird species may enhance its function, thereby limiting increases in body size. This phenomenon has also been investigated in cetaceans (Sun et al. 2019), potentially reflecting a similar constraining role in larger avian species.

Additionally, we found that the *IGF2BP1* gene has been widely under the positive selection across whole avian lineages of different body sizes. Notably, its evolutionary rate is positively correlated with body mass (Figure 4). Moreover, extreme‐sized birds exhibit a relatively strong positive selection, both the small‐bodied *Cyanistes caeruleus* and large‐bodied *Struthio camelus* (Figure 2; Table S6). This gene is crucial for embryonic development, tumor formation, and cellular regulation. By stabilizing and regulating various mRNAs, it influences cellular proliferation, differentiation, and stress responses (Weidensdorfer et al. 2009). These findings suggest that *IGF2BP1* is integral to the evolutionary dynamics of avian body size.

Further investigation revealed significant differences in positive selection sites among these genes. An in‐depth examination of functional information of these sites showed that they are located in important functional and structural domains (Table S7). For instance, positive selection sites in the *PLXDC2* gene are primarily found in large and medium‐sized birds. Specifically, in the large bird species *Aptenodytes patagonicus*, the 491 site of the PLXDC2 is located in the topology domain—the cytoplasmic region. This gene is primarily expressed in the endothelial cells of tumor vasculature (Cheng et al. 2014), potentially reflecting specific selective pressures related to large body size regulation. On the other side, the *ATP11A* gene shows specific site distribution in large‐ and medium‐sized birds, primarily concentrated in topology and transmembrane domains, which are crucial for its ATPase activity and membrane lipid transport functions (Ochiai et al. 2022). These findings suggest that mutations in functionally specific gene sites may be the fundamental cause of body size differences in birds.

When further exploring the impact of these genes on body size evolution in closely related species, we also found the significant differences in gene evolution within avian order, particularly in the unique adaptive patterns exhibited by Galliformes and Sphenisciformes (Figure 3A,B). In Galliformes, the *PLXDC2* and *ATP11A* genes are under significant selection pressure in large‐ and medium‐sized birds; selection sites in the topology domain of ATP11A might relate to energy metabolism needs for aquatic life (Chaubey et al. 2016), which indicated their key roles in size regulation. In *Gallus gallus*, glycosylation modification at the 160th site of the *PLXDC2* gene may be related to size characteristics (Cheng et al. 2014). In contrast, within Sphenisciformes, size‐related genes such as *GRB10* and *OBSL1* exhibit much stronger selection pressures. Previous researches have found that the evolution of these genes have been related to  short stature as well as experience similar selective pressures in large aquatic mammals, such as cetaceans  (Sun et al. 2019). This may reflect commonalities in the evolutionary processes that shape body size in aquatic animals. These findings reveal the unique adaptive patterns in size evolution in Galliformes and Sphenisciformes, demonstrating the diverse and complex impacts of ecological environments and gene selection pressures on size evolution.

### 4.2 Parallel/Convergent Evolution of Large Avian Species Lineages

Convergent evolution is defined as the process by which distinct species independently develop analogous characteristics or functions in response to similar environmental pressures (Losos 2011). This phenomenon represents a significant area of inquiry within evolutionary biology and has been documented across a diverse array of biological taxa (Rundell and Leander 2010; Mao et al. 2020; Kazandjian et al. 2021; Li et al. 2024). The driving forces behind convergent evolution include environmental factors such as climate change and habitat characteristics (Beasley, Jason, and Miller 2012; Jiang, Cowell, and Nakazawa 2013), as well as the physiological and genetic traits of the organisms (Stern 2013). At the molecular level, convergent evolution is often evidenced by variations in gene evolutionary rates (Chikina, Robinson, and Clark 2016), Gene Ontology (GO) or Kyoto Encyclopedia of Genes and Genomes (KEGG) enrichment of positively selected genes (Sun et al. 2018), and specific amino acid substitutions (Parker et al. 2013; Chai et al. 2020), among other indicators.

Convergent amino acid substitution is commonly recognized as a significant indicator of evolutionary adaptation, and it has been extensively utilized in contemporary research (Mu et al. 2021; Kang et al. 2023; Wang et al. 2024). Our investigation identified 196 convergent and parallel substitution sites across 12 genes, which are distributed among large‐bodied avian species. This suggests a widespread occurrence of convergent evolution in genes associated with body size within these lineages. Further analysis has indicated that these substitution sites are located within critical functional and structural domains. For instance, in the ACAN, amino acid substitutions predominantly occur near glycosylation sites (e.g., positions 328 and 337). The proteoglycan (ACAN) provides a hydrated gel structure in cartilage by binding hyaluronic acid, thereby playing a vital role in the interaction between cartilage and the extracellular matrix (Krueger, Kurima, and Schwartz 1999). The substitution at position 337 may influence disulfide bond formation, potentially affecting the integrity and mechanical properties of cartilage. In NCAPG, amino acid substitutions at positions 928 and 952, which reside in the Armadillo‐type fold domain, are crucial for protein interactions and cellular stability (Kim et al. 2014). In the OBSL1, amino acid substitutions at positions 18 and 79 likely enhance its stability and interaction with muscle proteins, thereby supporting muscle structure and function in large avian species (Hanson et al. 2011). Similarly, for the EIF2AK3, the parallel substitution positions at sites 550, 702, and 806 are located within the topological domain; these substitutions may optimize the localization and functionality of EIF2AK3, thereby enhancing its kinase activity and regulatory role in protein synthesis under stress conditions (Delépine et al. 2000). These findings suggest that convergent evolution in avian body size may be associated with the convergence of protein function. Previous studies have indicated that increases in body size are more closely related to genetic factors than to environmental conditions (Ruebenstahl et al. 2024). In this study, we examined the relationship between the convergent evolution of bird body size and genetic factors throughout evolutionary history. However, further investigation is required to determine whether additional genetic convergences, such as those previously mentioned, exist. This necessitates more comprehensive data, including genomic convergence, as well as additional research that integrates fossil evidence, feeding habits, metabolic processes, and various ecological guilds. Overall, our study highlights the significance of key convergent substitutions in genes related to body size adaptation, providing valuable insights into the mechanisms of convergent evolution in Aves.

### 4.3 Association Between Gene Evolution and Morphological Variables

The correlation between evolutionary rates and phenotypes significantly enhances our understanding of adaptive evolution in various species. The statistical association between the selection of functional genes and phenotypic changes is crucial for investigating the genetic basis of adaptive phenotypes (Montgomery, Mundy, and Barton 2014). Notably, we observed a significant positive correlation between body mass and the evolutionary rate of the *IGF2BP1* gene. Our findings suggest that this gene primarily functions to promote body size growth in large‐bodied bird species. As an RNA‐binding protein, IGF2BP1 regulates mRNA stability and translation by binding to N6‐methyladenosine (m6A)‐modified mRNA, thereby influencing neural development, cell migration, as well as muscle and skeletal development (Huang et al. 2018; Müller et al. 2020). These functions play a significant supportive role in the process of body shaping. Our results indicate that the evolution of avian body size has been driven by the selection of *IGF2BP1*. Furthermore, the selection signal of *IGF2BP1* is particularly strong across different avian lineages with varying body sizes (Table S6a), further underscoring the importance of the *IGF2BP1* gene in regulating avian body characteristics. This provides a deeper understanding of the evolutionary mechanisms underlying body size in birds.

## 5 Conclusions

Birds exhibit an exceptionally broad range of body sizes, which has been attributed to various factors. In this study, we first investigate the evolution of body size‐related genes in Aves. Our results confirm that body size‐related genes have evolved adaptively across avian lineages, demonstrating divergent selective patterns among different bodied‐size lineages. This pattern is also evident in smaller datasets (datasets 2 and 3). Based on dataset 1, *IGF2BP1* appears to play a crucial role in the evolution of body size in birds. Furthermore, accelerated evolutionary rates were identified in *IGFBP7* and *PLXDC2*. Notably, we report for the first time the convergent evolution of avian body size. These findings provide significant genetic data and insights for understanding the adaptive evolution of body size in birds.

## Conflicts of Interest

The authors declare no conflicts of interest.

## Supporting information



Figure S1 The phylogeny of species used in this study. Branches a‐l in the tree were used for the detection of convergent/parallel amino acid substitutions.

Table S1 The basic information of each species used in this study. a is for 56 species (dataset 1); b is for Galliformes (dataset 2); c is for Sphenisciformes (dataset 3)

Table S2 The information of candidate genes for this study

Table S3 The genes accession numbers of each species used in this study

Table S4 Results of the one‐ratio model analysis for each dataset. a is for 56 avian species; b is for Galliformes; c is for Sphenisciformes.

Table S5 The results of free ratio model for each dataset. a is for 56 avian species; b is for Galliformes; c is for Sphenisciformes

Table S6 The results of branch‐site model for each dataset. a is for 56 avian species; b is for Galliformes; c is for Sphenisciformes

Table S7 The annotation information of functional sites and domains of branch‐site model analysis for each dataset (identity with sites of human). a is for 56 avian species; b is for Galliformes; c is for Sphenisciformes

Table S8 The results of parallel/convergent amino acid substitution sites

Table S9 The annotation information of functional sites and domains of parallel/convergent analysis (identity with sites of human)

Table S10 Phylogenetic generalized least squares (PGLS) regression analyses of gene evolutionary rates and body mass
