# Supplementary figures and images for: Insight Into Body Size Evolution in Aves: Based on Some Body Size‐Related Genes
Source: Integr Zool. 2024 Dec 11;20(6):1124–35. doi: 10.1111/1749-4877.12927 (PMC12618961; doi:10.1111/1749-4877.12927)

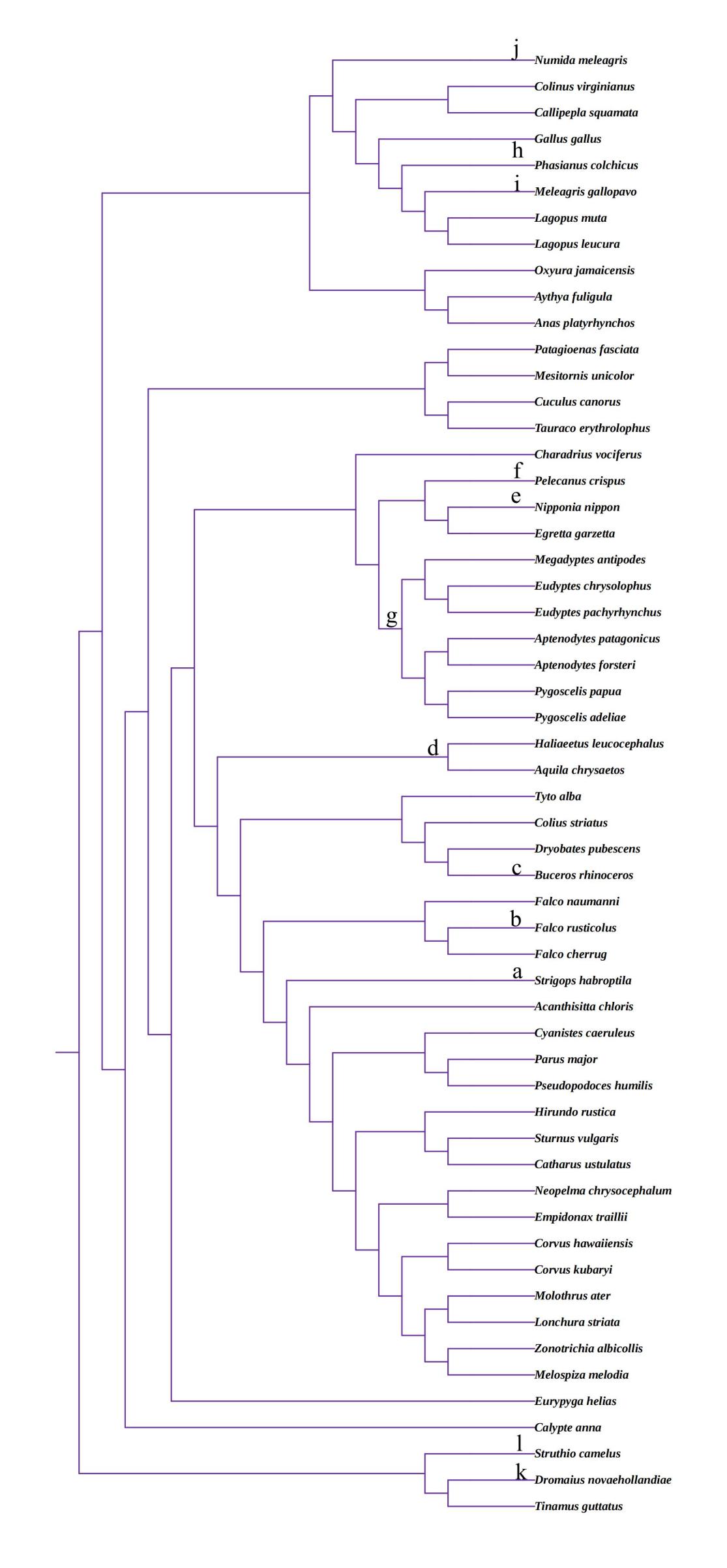

Supplement: Supplementary file 1 — Figure S1 The phylogeny of species used in this study. Branches a‐l in the tree were used for the detection of convergent/parallel amino acid substitutions. [file INZ2-20-1124-s001.docx]
