# Supplementary material for: Insight Into Body Size Evolution in Aves: Based on Some Body Size‐Related Genes
Source: Integr Zool. 2024 Dec 11;20(6):1124–35. doi: 10.1111/1749-4877.12927 (PMC12618961; doi:10.1111/1749-4877.12927)
Supplement: Supplementary file 2 — Table S1 The basic information of each species used in this study. a is for 56 species (dataset 1); b is for Galliformes (dataset 2); c is for Sphenisciformes (dataset 3) [file INZ2-20-1124-s008.docx]

**Table S2a** The basic information for 56 avian species.

| **Species** | **Family** | **Order** | **Mass(g)** |
| --- | --- | --- | --- |
| *Struthio camelus* | Struthionidae | Struthioniformes | 111000.0 |
| *Dromaius novaehollandiae* | Casuariidae | Struthioniformes | 34093.3 |
| *Aptenodytes forsteri* | Spheniscidae | Sphenisciformes | 33569.3 |
| *Aptenodytes patagonicus* | Spheniscidae | Sphenisciformes | 11731.1 |
| *Pelecanus crispus* | Pelecanidae | Pelecaniformes | 9512.1 |
| *Pygoscelis papua* | Spheniscidae | Sphenisciformes | 5933.0 |
| *Meleagris gallopavo* | Phasianidae | Galliformes | 5791.4 |
| *Megadyptes antipodes* | Spheniscidae | Sphenisciformes | 5326.2 |
| *Pygoscelis adeliae* | Spheniscidae | Sphenisciformes | 4847.7 |
| *Haliaeetus leucocephalus* | Accipitridae | Accipitriformes | 4700.6 |
| *Eudyptes chrysolophus* | Spheniscidae | Sphenisciformes | 4485.1 |
| *Aquila chrysaetos* | Accipitridae | Accipitriformes | 4248.0 |
| *Eudyptes pachyrhynchus* | Spheniscidae | Sphenisciformes | 3904.9 |
| *Buceros rhinoceros* | Bucerotidae | Bucerotiformes | 2371.6 |
| *Nipponia nippon* | Threskiornithidae | Pelecaniformes | 1900.0 |
| *Strigops habroptila* | Strigopidae | Psittaciformes | 1732.1 |
| *Falco rusticolus* | Falconidae | Falconiformes | 1431.7 |
| *Numida meleagris* | Numididae | Galliformes | 1299.0 |
| *Phasianus colchicus* | Phasianidae | Galliformes | 1120.3 |
| *Falco cherrug* | Falconidae | Falconiformes | 961.2 |
| *Anas platyrhynchos* | Anatidae | Anseriformes | 843.4 |
| *Gallus gallus* | Phasianidae | Galliformes | 751.7 |
| *Aythya fuligula* | Anatidae | Anseriformes | 701.2 |
| *Tinamus guttatus* | Tinamidae | Struthioniformes | 686.2 |
| *Oxyura jamaicensis* | Anatidae | Anseriformes | 608.2 |
| *Lagopus muta* | Phasianidae | Galliformes | 535.3 |
| *Corvus hawaiiensis* | Corvidae | Passeriformes | 518.8 |
| *Tyto alba* | Tytonidae | Strigiformes | 403.3 |
| *Patagioenas fasciata* | Columbidae | Columbiformes | 366.3 |
| *Lagopus leucura* | Phasianidae | Galliformes | 355.0 |
| *Egretta garzetta* | Ardeidae | Pelecaniformes | 312.0 |
| *Tauraco erythrolophus* | Musophagidae | Musophagiformes | 261.2 |
| *Corvus kubaryi* | Corvidae | Passeriformes | 249.9 |
| *Eurypyga helias* | Eurypygidae | Eurypygiformes | 210.0 |
| *Callipepla squamata* | Odontophoridae | Galliformes | 183.9 |
| *Colinus virginianus* | Odontophoridae | Galliformes | 171.9 |
| *Falco naumanni* | Falconidae | Falconiformes | 152.1 |
| *Mesitornis unicolor* | Mesitornithidae | Mesitornithiformes | 148.0 |
| *Cuculus canorus* | Cuculidae | Cuculiformes | 111.4 |
| *Charadrius vociferus* | Charadriidae | Charadriiformes | 96.4 |
| *Sturnus vulgaris* | Sturnidae | Passeriformes | 77.1 |
| *Colius striatus* | Coliidae | Coliiformes | 51.1 |
| *Pseudopodoces humilis* | Paridae | Passeriformes | 45.4 |
| *Molothrus ater* | Icteridae | Passeriformes | 40.3 |
| *Catharus ustulatus* | Turdidae | Passeriformes | 30.3 |
| *Dryobates pubescens* | Picidae | Piciformes | 25.6 |
| *Zonotrichia albicollis* | Passerellidae | Passeriformes | 24.4 |
| *Melospiza melodia* | Passerellidae | Passeriformes | 21.9 |
| *Hirundo rustica* | Hirundinidae | Passeriformes | 17.9 |
| *Parus major* | Paridae | Passeriformes | 16.3 |
| *Neopelma chrysocephalum* | Pipridae | Passeriformes | 15.5 |
| *Empidonax traillii* | Tyrannidae | Passeriformes | 13.4 |
| *Lonchura striata* | Estrildidae | Passeriformes | 12.3 |
| *Cyanistes caeruleus* | Paridae | Passeriformes | 11.1 |
| *Acanthisitta chloris* | Acanthisittidae | Passeriformes | 6.9 |
| *Calypte anna* | Trochilidae | Caprimulgiformes | 4.2 |

**Table S2b** The basic information for Galliformes

| **Species** | **Family** | **Order** | **Mass(g)** |
| --- | --- | --- | --- |
| *Coturnix japonica* | Phasianidae | Galliformes | 94.8 |
| *Colinus virginianus* | Odontophoridae | Galliformes | 171.9 |
| *Callipepla squamata* | Odontophoridae | Galliformes | 183.9 |
| *Lagopus leucura* | Phasianidae | Galliformes | 355.0 |
| *Lagopus muta* | Phasianidae | Galliformes | 535.3 |
| *Gallus gallus* | Phasianidae | Galliformes | 751.7 |
| *Phasianus colchicus* | Phasianidae | Galliformes | 1120.3 |
| *Numida meleagris* | Numididae | Galliformes | 1299.0 |
| *Centrocercus urophasianus* | Phasianidae | Galliformes | 1902.5 |
| *Meleagris gallopavo* | Phasianidae | Galliformes | 5791.4 |

**Table S2c** The basic information for Sphenisciformes.

| **Species** | **Family** | **Order** | **Mass(g)** |
| --- | --- | --- | --- |
| *Eudyptula minor* | Spheniscidae | Sphenisciformes | 1107.8 |
| *Spheniscus mendiculus* | Spheniscidae | Sphenisciformes | 1921.9 |
| *Eudyptes chrysocome* | Spheniscidae | Sphenisciformes | 2327.9 |
| *Spheniscus demersus* | Spheniscidae | Sphenisciformes | 3130.1 |
| *Eudyptes pachyrhynchus* | Spheniscidae | Sphenisciformes | 3904.9 |
| *Spheniscus magellanicus* | Spheniscidae | Sphenisciformes | 4105.1 |
| *Pygoscelis adeliae* | Spheniscidae | Sphenisciformes | 4847.7 |
| *Megadyptes antipodes* | Spheniscidae | Sphenisciformes | 5326.2 |
| *Pygoscelis papua* | Spheniscidae | Sphenisciformes | 5933.0 |
| *Aptenodytes patagonicus* | Spheniscidae | Sphenisciformes | 11731.1 |
| *Aptenodytes forsteri* | Spheniscidae | Sphenisciformes | 33569.3 |
