# Supplementary material for: Insight Into Body Size Evolution in Aves: Based on Some Body Size‐Related Genes
Source: Integr Zool. 2024 Dec 11;20(6):1124–35. doi: 10.1111/1749-4877.12927 (PMC12618961; doi:10.1111/1749-4877.12927)
Supplement: Supplementary file 3 — Table S2 The information of candidate genes for this study [file INZ2-20-1124-s005.docx]

**Table S2** The information of candidate genes for this study.

| **Gene** | **Protein Product** | **Function** | **Diseases** | References |
| --- | --- | --- | --- | --- |
| **Tall stature-related genes** | | | | |
| ***NCAPG*** | Non-SMC Condensin I Complex Subunit G | It has been reported to be linked to birth weight, withers height, feeding efciency, and pubertal growth. | The gene strongly associated with increased body size and weight gain. | Silva et al. 2023 |
| ***PLAG1*** | pleomorphic adenoma  gene 1 | Involved in cell proliferation and differentiation | PLAG1 KO phenotype is reduced body size | Juma et al. 2016 |
| ***EIF2AK3*** | translation initiation factor  2-α kinase 3 | Functions in maintaining the integrity of pancreatic β-cells, regulate protein translation | Wolcott-Rallison syndrome, growth retardation | Gupta et al. 2010 |
| ***GALNS*** | N-acetylgalactosamine-6-  sulfate-sulfatase | Derived from a cysteine residue by action of the formylglycine- generating enzyme | Morquio A disease, bone dysplasia, short trunk dwarfism | Tomatsu et al. 2010 |
| ***PLOD1*** | lysyl hydroxylase | Be responsible for hydroxylation of lysyl residues in collagen proteins | Nevo syndrome, tall stature | Visser et al. 2009 |
| **Short stature-related genes** | | | | |
| ***GRB10*** | growth factor receptor –  bound protein 10 | Mediates interactions between disparate proteins, role as a growth suppressor | Loss of Grb10 function in the mouse results in fetal and placental overgrowth, overexpression of GRB10 results in SRS | Charalambous et al. 2003 |
| ***ACAN*** | aggrecan | Be essential for cartilage structure | Bone development abnormalities in otherspecies, causing disproportionate dwarfism | Cavanagh et al. 2007 |
| ***OBSL1*** | Obscurin-like 1 | Functions as a cytoskeletal adaptor protein linking the nuclear proteins to the cytoplasmic support network | 3-M syndrome, body growth restriction. | Demir et al. 2013 |
| **Growth hormone/insulin-like growth factor axis** | | | | |
| ***GHSR*** | Growth Hormone Secretagogue Receptor | It is associated with the secretion of gastric acid, control of cell proliferation, apoptosis, lactation, and cardiovascular pressure. | This gene has been linked to  increased body size. | Silva et al. 2023 |
| ***IGFBP7*** | Insulin-Like Growth Factor Binding Protein 7 | *IGFBP7* is a 27 kD protein and a member of the IGFBP superfamily, responsible for the viability of insulinlike growth factors (IGFs)—molecules involved in promoting cell growth and d ivision. | In prostate, breast, lung, and colorectal cancer due to its regulatory action related to cell proliferation, cell adhesion, cell senescence, and angiogenesis | Silva et al. 2023 |
| **Body-size-related genes in birds** | | | | |
| ***ATP11A*** | ATPase phospholipid transporting 11A | This gene involved in metabolism in duck fat cells, involved in  basic growth and development processes, especially in  relation to fat deposition. | Transporting creates membrane phospholipid asymmetry and initiates the biogenesis of transport vesicles. | Deng et al. 2019 |
| ***PLDXC2*** | Plexin domain containing 2 | This gene was coordinates the development and differentiation of nerve cells in various animals. | This gene was associated with both body weight and leg muscle weight. | Deng et al. 2019 |
| ***TUBGCP3*** | Encoding tubulin gamma complex-associated protein 3 | This gene was associated with four traits (42-day body weight, eviscerated weight, half-eviscerated weight, and leg muscle weight percentage). |  | Deng et al. 2019 |
| ***TNS3*** | Tensin 3 | *TNS3* genes have been shown to have a synergistic effect on the maintenance of osteoclast activity  to ensure the correct organization of podosomes. | This gene was associated with fossil bone length. QTL information indicated that the QTL related to body weight and breast muscle weight/percentage localized to *TNS3*. | Deng et al. 2019 |
| ***IGF2BP1*** | insulin-like growth factor II mRNA binding protein 1 | *IGF2BP1* belongs to a family of RNA-binding proteins that are  implicated in mRNA localization, turnover, and translational  control. | *IGF2BP1*-deﬁcient mice show dwarﬁsm, impaired gut development, and down regulation of IGF2 expression at the embryonic stage. | Deng et al. 2019 |

# References

Cavanagh J A L, Tammen I, Windsor P A, et al. Bulldog dwarfism in Dexter cattle is caused by mutations in ACAN. *Mammalian Genome*, **2007**, 18: 808-814.

Charalambous M, Smith F M, Bennett W R, et al. Disruption of the imprinted Grb10 gene leads to disproportionate overgrowth by an Igf2-independent mechanism. *Proceedings of the National Academy of Sciences*, **2003**, 100(14): 8292-8297.

Deng M T, Zhu F, Yang Y Z, et al. Genome-wide association study reveals novel loci associated with body size and carcass yields in Pekin ducks. *BMC Genomics*, **2019**, 20: 1-13.

Gupta S, McGrath B, Cavener D R. PERK (EIF2AK3) regulates proinsulin trafficking and quality control in the secretory pathway. *Diabetes*, **2010**, 59(8): 1937-1947.

Juma A R, Damdimopoulou P E, Grommen S V H, et al. Emerging role of PLAG1 as a regulator of growth and reproduction. *Journal of Endocrinology*, **2016**, 228(2): R45-R56.

Sun Y, Liu Y, Sun X, Lin Y, Yin D, Xu S, & Yang G. (2019). Insights into body size variation in cetaceans from the evolution of body-size-related genes. *BMC Evolutionary Biology*, **19**, 1-11.

Silva F A, Souza É M S, Ramos E, et al. The molecular evolution of genes previously associated with large sizes reveals possible pathways to cetacean gigantism. *Scientific Reports*, **2023**, 13(1): 67.

Tomatsu S, Montaño A M, Nishioka T, et al. Mutation and polymorphism spectrum of the GALNS gene in mucopolysaccharidosis IVA (Morquio A). *Human Mutation*, **2005**, 26(6): 500-512.

Visser R, Kant S G, Wit J M, et al. Overgrowth syndromes: from classical to new. Pediatr Endocrinol Rev, 2009, 6(3): 375-394.

Zhou Z, Li M, Cheng H, et al. An intercross population study reveals genes associated with body size and plumage color in ducks. *Nature Communications*, **2018**, 9(1): 2648.
