# Supplementary material for: Insight Into Body Size Evolution in Aves: Based on Some Body Size‐Related Genes
Source: Integr Zool. 2024 Dec 11;20(6):1124–35. doi: 10.1111/1749-4877.12927 (PMC12618961; doi:10.1111/1749-4877.12927)
Supplement: Supplementary file 5 — Table S4 Results of the one‐ratio model analysis for each dataset. a is for 56 avian species; b is for Galliformes; c is for Sphenisciformes. [file INZ2-20-1124-s002.docx]

**Table S4a** Results of the one-ratio model analysis for 56 avian species.

| **Genes** | **Models** | **-InL** | **2∆InL** | ***P* value** | **ω values** |
| --- | --- | --- | --- | --- | --- |
| *ACAN* | One-ratio | 67274.126450 | 5052.02474 | 0 | 0.25177 |
|  | One-ratio=1 | 69800.138822 |  |  | 1 |
| *EIF2AK3* | One-ratio | 26465.068125 | 4154.74679 | 0 | 0.10755 |
|  | One-ratio=1 | 28542.441518 |  |  | 1 |
| *GALNS* | One-ratio | 11202.376354 | 1774.41853 | 0 | 0.11583 |
|  | One-ratio=1 | 12089.585618 |  |  | 1 |
| *GHSR* | One-ratio | 7166.066743 | 2072.4323 | 0 | 0.05371 |
|  | One-ratio=1 | 8202.282894 |  |  | 1 |
| *GRB10* | One-ratio | 8116.209867 | 1384.32785 | 5.3461E-303 | 0.06051 |
|  | One-ratio=1 | 8808.373792 |  |  | 1 |
| *IGFBP7* | One-ratio | 4520.873283 | 550.262808 | 1.1036E-121 | 0.14172 |
|  | One-ratio=1 | 4796.004687 |  |  | 1 |
| *NCAPG* | One-ratio | 34663.866099 | 1418.53583 | 1.9705E-310 | 0.34313 |
|  | One-ratio=1 | 35373.134012 |  |  | 1 |
| *OBSL1* | One-ratio | 68853.265983 | 11735.8825 | 0 | 0.12796 |
|  | One-ratio=1 | 74721.207235 |  |  | 1 |
| *PLAG1* | One-ratio | 4732.593126 | 799.027072 | 8.782E-176 | 0.02891 |
|  | One-ratio=1 | 5132.106662 |  |  | 1 |
| *PLOD1* | One-ratio | 16370.764016 | 2273.76561 | 0 | 0.12489 |
|  | One-ratio=1 | 17507.646821 |  |  | 1 |
| *ATP11A* | One-ratio | 21269.995958 | 1116.98508 | 6.7189E-245 | 0.13285 |
|  | One-ratio=1 | 21828.488498 |  |  |  |
| *PLXDC2* | One-ratio | 10395.462496 | 540.229488 | 1.6808E-119 | 0.14019 |
|  | One-ratio=1 | 10665.577240 |  |  | 1 |
| *TNS3* | One-ratio | 44029.370566 | 335.126844 | 7.34818E-75 | 0.31784 |
|  | One-ratio=1 | 44196.933988 |  |  | 1 |
| *TUBGCP3* | One-ratio | 18401.307800 | 1905.999032 | 0 | 0.07239 |
|  | One-ratio=1 | 19354.307316 |  |  | 1 |
| *IGF2BP1* | One-ratio | 15510.966677 | 3974.370814 | 0 | 0.07180 |
|  | One-ratio=1 | 17498.152177 |  |  |  |

**Table S4b** Results of the one-ratio model analysis for Galliformes.

| **Genes** | **Models** | **-InL** | **2∆InL** | ***P* value** | **ω values** |
| --- | --- | --- | --- | --- | --- |
| *ACAN* | One-ratio | 15293.289095 | 223.58188 | 1.49662E-50 | 0.20428 |
|  | One-ratio=1 | 15405.080035 |  |  |  |
| *EIF2AK3* | One-ratio | 7400.548378 | 243.191188 | 7.92324E-55 | 0.11360 |
|  | One-ratio=1 | 7522.143972 |  |  |  |
| *GALNS* | One-ratio | 3667.472816 | 81.139304 | 2.10354E-19 | 0.15904 |
|  | One-ratio=1 | 3708.042468 |  |  |  |
| *GHSR* | One-ratio | 2399.141305 | 123.851576 | 9.07868E-29 | 0.08711 |
|  | One-ratio=1 | 2461.067093 |  |  |  |
| *GRB10* | One-ratio | 3346.891339 | 78.762522 | 7.00433E-19 | 0.09614 |
|  | One-ratio=1 | 3386.272600 |  |  |  |
| *IGFBP7* | One-ratio | 3721.844667 | 289.27342 | 7.15933E-65 | 0.03756 |
|  | One-ratio=1 | 3866.481377 |  |  |  |
| *NCAPG* | One-ratio | 8553.848261 | 140.275442 | 2.3173E-32 | 0.20855 |
|  | One-ratio=1 | 8623.985982 |  |  |  |
| *OBSL1* | One-ratio | 17668.200942 | 590.286232 | 2.1709E-130 | 0.14741 |
|  | One-ratio=1 | 17963.344058 |  |  |  |
| *PLAG1* | One-ratio | 2398.487912 | 59.83745 | 1.03024E-14 | 0.02477 |
|  | One-ratio=1 | 2428.406637 |  |  |  |
| *PLOD1* | One-ratio | 4781.704934 | 185.480354 | 3.08271E-42 | 0.08930 |
|  | One-ratio=1 | 4874.445111 |  |  |  |
| *IGFBP7* | One-ratio | 2383.990148 | 15.44025 | 8.51551E-05 | 0.28731 |
|  | One-ratio=1 | 2391.710273 |  |  |  |
| *ATP11A* | One-ratio | 8390.743906 | 84.987974 | 3.00185E-20 | 0.22970 |
|  | One-ratio=1 | 8433.237893 |  |  |  |
| *PLXDC2* | One-ratio | 4353.408563 | 9.504614 | 0.002049559 | 0.36103 |
|  | One-ratio=1 | 4358.160870 |  |  |  |
| *TNS3* | One-ratio | 11453.900777 | 2.126002 | 0.144818048 | 0.37002 |
|  | One-ratio=1 | 11452.837776 |  |  |  |
| *TUBGCP3* | One-ratio | 5504.081807 | 283.882802 | 1.07025E-63 | 0.04972 |
|  | One-ratio=1 | 5646.023208 |  |  |  |

**Table S4c** Results of the one-ratio model analysis for Sphenisciformes.

| **Genes** | **Models** | **-InL** | **2∆InL** | ***P* value** | **ω values** |
| --- | --- | --- | --- | --- | --- |
| *ACAN* | One-ratio | 11834.803714 | 45.663916 | 1.40385E-11 | 0.26539 |
|  | One-ratio=1 | 11857.635672 |  |  |  |
| *EIF2AK3* | One-ratio | 4892.806925 | 62.951602 | 2.11849E-15 | 0.09128 |
|  | One-ratio=1 | 4924.282726 |  |  |  |
| *GALNS* | One-ratio | 2422.576218 | 19.282104 | 1.12758E-05 | 0.13026 |
|  | One-ratio=1 | 2432.217270 |  |  |  |
| *GHSR* | One-ratio | 2866.334154 | 120.287868 | 5.47157E-28 | 0.08163 |
|  | One-ratio=1 | 2926.478088 |  |  |  |
| *GRB10* | One-ratio | 4331.902215 | 0.585806 | 0.444045511 | 0.55127 |
|  | One-ratio=1 | 4332.195118 |  |  |  |
| *NCAPG* | One-ratio | 5005.193885 | 4.033868 | 0.044595584 | 0.34221 |
|  | One-ratio=1 | 5007.210819 |  |  |  |
| *OBSL1* | One-ratio | 12739.684471 | 182.171658 | 1.62658E-41 | 0.19905 |
|  | One-ratio=1 | 12830.770300 |  |  |  |
| *PLAG1* | One-ratio | 2110.989580 | 15.2897 | 9.2218E-05 | 0.00010 |
|  | One-ratio=1 | 2118.634430 |  |  |  |
| *PLOD1* | One-ratio | 2088.531448 | 11.824478 | 0.000584571 | 0.12557 |
|  | One-ratio=1 | 2094.443687 |  |  |  |
| *ATP11A* | One-ratio | 5491.570317 | 76.392854 | 2.32491E-18 | 0.06883 |
|  | One-ratio=1 | 5529.766744 |  |  |  |
| *PLXDC2* | One-ratio | 2271.988195 | 22.940344 | 1.67108E-06 | 0.07983 |
|  | One-ratio=1 | 2283.458367 |  |  |  |
