# Supplementary material for: Insight Into Body Size Evolution in Aves: Based on Some Body Size‐Related Genes
Source: Integr Zool. 2024 Dec 11;20(6):1124–35. doi: 10.1111/1749-4877.12927 (PMC12618961; doi:10.1111/1749-4877.12927)
Supplement: Supplementary file 6 — Table S5 The results of free ratio model for each dataset. a is for 56 avian species; b is for Galliformes; c is for Sphenisciformes [file INZ2-20-1124-s007.docx]

**Table S5a** The results of free ratio model for the 56 avian species.

| **Genes** | **Model** | **-lnL** | **Model**  **comparison** | **2ΔlnL** | **df** | ***P*-value** |
| --- | --- | --- | --- | --- | --- | --- |
| *ACAN* | M0(one ratio) | 66990.460679 | M1vsM0 | 190.657932 | 109 | 2.17472E-06 |
|  | M1(free ratio) | 66990.460679 |  |  |  |  |
| *EIF2AK3* | M0(one ratio) | 24606.968081 | M1vsM0 | 173.357282 | 109 | 8.65001E-05 |
|  | M1(free ratio) | 24520.289440 |  |  |  |  |
| *GALNS* | M0(one ratio) | 11080.019696 | M1vsM0 | 149.88472 | 105 | 0.002668201 |
|  | M1(free ratio) | 11005.075986 |  |  |  |  |
| *GHSR* | M0(one ratio) | 7103.717249 | M1vsM0 | 146.805584 | 105 | 0.004440275 |
|  | M1(free ratio) | 7030.314457 |  |  |  |  |
| *GRB10* | M0(one ratio) | 8000.662493 | M1vsM0 | 0.191372656 | 115 | 0.191372656 |
|  | M1(free ratio) | 7942.998875 |  |  |  |  |
| *NCAPG* | M0(one ratio) | 34569.808811 | M1vsM0 | 245.847336 | 109 | 1.43837E-12 |
|  | M1(free ratio) | 34446.885143 |  |  |  |  |
| *PLAG1* | M0(one ratio) | 4702.129276 | M1vsM0 | 59555.4956 | 109 | 0 |
|  | M1(free ratio) | 4669.137324 |  |  |  |  |
| *PLOD1* | M0(one ratio) | 16174.682755 | M1vsM0 | 111.865232 | 107 | 0.354539965 |
|  | M1(free ratio) | 16118.750139 |  |  |  |  |
| *OBSL1* | M0(one ratio) | 68653.200971 | M1vsM0 | 249.813722 | 93 | 2.7166E-16 |
|  | M1(free ratio) | 68528.294110 |  |  |  |  |
| *IGFBP7* | M0(one ratio) | 4488.487303 | M1vsM0 | 107.877344 | 97 | 0.211570227 |
|  | M1(free ratio) | 4434.548631 |  |  |  |  |
| *TUBGCP3* | M0(one ratio) | 18401.307800 | M1vsM0 | 515.179962 | 105 | 2.19626E-55 |
|  | M1(free ratio) | 18143.717819 |  |  |  |  |
| *TNS3* | M0(one ratio) | 44029.370566 | M1vsM0 | 398.373832 | 99 | 1.55148E-37 |
|  | M1(free ratio) | 43830.183650 |  |  |  |  |
| *PLXDC2* | M0(one ratio) | 10395.462496 | M1vsM0 | 252.058698 | 90 | 2.79303E-17 |
|  | M1(free ratio) | 10269.433147 |  |  |  |  |
| *ATP11A* | M0(one ratio) | 21269.995958 | M1vsM0 | 367.991562 | 99 | 1.3449E-32 |
|  | M1(free ratio) | 21086.000177 |  |  |  |  |

**Table S5b** The results of free ratio model for the Galliformes.

| **Gene** | **Model** | **-lnL** | **Model**  **comparison** | **2ΔlnL** | **df** | ***P*-value** |
| --- | --- | --- | --- | --- | --- | --- |
| *EIF2AK3* | M0(one ratio) | 7400.548378 | M1vsM0 | 31.655072 | 17 | 0.016602769 |
|  | M1(free ratio) | 7384.720842 |  |  |  |  |
| *GALNS* | M0(one ratio) | 3667.472816 | M1vsM0 | 45.1743 | 17 | 0.000229375 |
|  | M1(free ratio) | 3644.885666 |  |  |  |  |
| *IGFBP7* | M0(one ratio) | 2383.990148 | M1vsM0 | 31.67719 | 13 | 0.002681425 |
|  | M1(free ratio) | 2368.151553 |  |  |  |  |
| *NCAPG* | M0(one ratio) | 8553.848261 | M1vsM0 | 35.488074 | 17 | 0.005364431 |
|  | M1(free ratio) | 8536.104224 |  |  |  |  |
| *OBSL1* | M0(one ratio) | 17668.200942 | M1vsM0 | 39.660862 | 13 | 0.000156721 |
|  | M1(free ratio) | 17648.37051 |  |  |  |  |
| *ACAN* | M0(one ratio) | 15293.2891 | M1vsM0 | 9.102564 | 17 | 0.936989042 |
|  | M1(free ratio) | 15288.73781 |  |  |  |  |
| *GHSR* | M0(one ratio) | 2399.141305 | M1vsM0 | 25.847104 | 17 | 0.077291546 |
|  | M1(free ratio) | 2386.217753 |  |  |  |  |
| *GRB10* | M0(one ratio) | 3346.891339 | M1vsM0 | 57.91218 | 17 | 2.31357E-06 |
|  | M1(free ratio) | 3317.935249 |  |  |  |  |
| *IGF2BP1* | M0(one ratio) | 3721.844667 | M1vsM0 | 101.078738 | 17 | 5.61251E-14 |
|  | M1(free ratio) | 3671.305298 |  |  |  |  |
| *PLAG1* | M0(one ratio) | 2398.487912 | M1vsM0 | 7.21952 | 17 | 0.980523998 |
|  | M1(free ratio) | 2394.878152 |  |  |  |  |
| *PLOD1* | M0(one ratio) | 4781.704934 | M1vsM0 | 17.527864 | 17 | 0.419193839 |
|  | M1(free ratio) | 4772.941002 |  |  |  |  |
| *ATP11A* | M0(one ratio) | 8390.743906 | M1vsM0 | 259.746776 | 17 | 2.12095E-45 |
|  | M1(free ratio) | 8260.870518 |  |  |  |  |
| *PLXDC2* | M0(one ratio) | 4353.408563 | M1vsM0 | 114.800024 | 15 | 1.9206E-17 |
|  | M1(free ratio) | 4296.008551 |  |  |  |  |
| *TNS3* | M0(one ratio) | 11453.900777 | M1vsM0 | 55.589716 | 17 | 5.50791E-06 |
|  | M1(free ratio) | 11426.105919 |  |  |  |  |
| *TUBGCP3* | M0(one ratio) | 5504.081807 | M1vsM0 | 11844.04822 | 17 | 0 |
|  | M1(free ratio) | 5477.223644 |  |  |  |  |

**Table S5c** The results of free ratio model for the Sphenisciformes.

| **Gene** | **Model** | **-lnL** | **Model**  **comparison** | **2ΔlnL** | **df** | ***P*-value** |
| --- | --- | --- | --- | --- | --- | --- |
| *ACAN* | M0(one ratio) | 11834.803714 | M1vsM0 | 13.068324 | 17 | 0.731600268 |
|  | M1(free ratio) | 11828.269552 |  |  |  |  |
|  |  |  |  |  |  |  |
| *EIF2AK3* | M0(one ratio) | 4892.806925 | M1vsM0 | 25.547722 | 19 | 0.143291997 |
|  | M1(free ratio) | 4880.033064 |  |  |  |  |
| *GALNS* | M0(one ratio) | 2422.576218 | M1vsM0 | 13.242072 | 19 | 0.82595133 |
|  | M1(free ratio) | 2415.955182 |  |  |  |  |
| *GHSR* | M0(one ratio) | 2866.334154 | M1vsM0 | 37.764982 | 19 | 0.006356816 |
|  | M1(free ratio) | 2847.451663 |  |  |  |  |
| *GRB10* | M0(one ratio) | 4331.902215 | M1vsM0 | 47.569984 | 19 | 0.00029588 |
|  | M1(free ratio) | 4308.117223 |  |  |  |  |
| *NCAPG* | M0(one ratio) | 5005.193885 | M1vsM0 | 21.082416 | 19 | 0.332264905 |
|  | M1(free ratio) | 4994.652677 |  |  |  |  |
| *OBSL1* | M0(one ratio) | 12739.684471 | M1vsM0 | 62.582056 | 19 | 1.50053E-06 |
|  | M1(free ratio) | 12708.393443 |  |  |  |  |
| *PLAG1* | M0(one ratio) | 2110.989580 | M1vsM0 | 0.000408 | 19 | 1 |
|  | M1(free ratio) | 2110.989784 |  |  |  |  |
| *PLOD1* | M0(one ratio) | 2088.531448 | M1vsM0 | 6.534756 | 19 | 0.996311461 |
|  | M1(free ratio) | 2085.264070 |  |  |  |  |
| *ATP11A* | M0(one ratio) | 5491.570317 | M1vsM0 | 19.78741 | 19 | 0.407471114 |
|  | M1(free ratio) | 5481.676612 |  |  |  |  |
| *PLXDC2* | M0(one ratio) | 2271.988195 | M1vsM0 | 10.654044 | 19 | 0.93492791 |
|  | M1(free ratio) | 2266.661173 |  |  |  |  |
