# Supplementary material for: Insight Into Body Size Evolution in Aves: Based on Some Body Size‐Related Genes
Source: Integr Zool. 2024 Dec 11;20(6):1124–35. doi: 10.1111/1749-4877.12927 (PMC12618961; doi:10.1111/1749-4877.12927)
Supplement: Supplementary file 7 — Table S6 The results of branch‐site model for each dataset. a is for 56 avian species; b is for Galliformes; c is for Sphenisciformes [file INZ2-20-1124-s006.docx]

**Table S6a** The results of branch-site model for 56 avian species.

| **Genes** | **The branch of** | **Model** | **-lnL** | **Model comparison** | **2ΔlnL** | **df** | ***P*-value** | **Parameters** | **Positive site (PP≥80%)** |
| --- | --- | --- | --- | --- | --- | --- | --- | --- | --- |
| *ACAN* | *Colius striatus* | Ma | -65553.717238 | Ma vs Ma0 | 7.235712 | 1 | 0.007146746 | ω0 = 0.09533ω1 = 1.00000ω2 = 14.33187 | 26 S 0.852  910 S 0.821  1227 G 0.847 |
|  |  | Ma0 | -65557.335094 |  |  |  |  | ω0 = 0.09523ω1 = 1.00000ω2 = 1.00000 |  |
| *ACAN* | *Tauraco erythrolophus* | Ma | -65549.162789 | Ma vs Ma0 | 17.229978 | 1 | 3.31169E-05 | ω0 = 0.09544ω1 = 1.00000ω2 = 582.11061 | 680 P 0.854  1877 - 0.859 |
|  |  | Ma0 | -65557.777778 |  |  |  |  | ω0 = 0.09539ω1 = 1.00000ω2 = 1.00000 |  |
| *ACAN* | *Melospiza melodia* | Ma | -65544.153627 | Ma vs Ma0 | 19.391808 | 1 | 1.06463E-05 | ω0 = 0.09506ω1 = 1.00000ω2 = 367.56052 | 663 C 0.942  664 Y 0.940 |
|  |  | Ma0 | -65553.849531 |  |  |  |  | ω0 = 0.09539ω1 = 0.09502ω2 = 1.00000 |  |
| *ACAN* | *Tinamus guttatus* | Ma | -65549.641409 | Ma vs Ma0 | 4.969148 | 1 | 0.025803355 | ω0 = 0.09546ω1 = 1.00000ω2 = 34.90085 | 724 E 0.850  2102 R 0.858 |
|  |  | Ma0 | -65552.125983 |  |  |  |  | ω0 = 0.09421ω1 = 1.00000ω2 = 1.00000 |  |
| *ACAN* | *Dromaius novaehollandiae* | Ma | -65553.163643 | Ma vs Ma0 | 4.20072 | 1 | 0.04040682 | ω0 = 0.09517ω1 = 1.00000ω2 = 13.91956 | 1586 F 0.866  1812 E 0.850 |
|  |  | Ma0 | -65555.264003 |  |  |  |  | ω0 = 0.09488ω1 = 1.00000ω2 = 1.00000 |  |
| *EIF2AK3* | *Parus major* | Ma | -24128.228545 | Ma vs Ma0 | 5.678344 | 1 | 0.017175571 | ω0 = 0.0.03430ω1 = 1.00000ω2 = 102.09362 | 826 S0.992** |
|  |  | Ma0 | -24131.067717 |  |  |  |  | ω0 = 0.03427ω1 = 1.00000ω2 = 1.00000 |  |
| *EIF2AK3* | *Cuculus canorus* | Ma | -24122.353259 | Ma vs Ma0 | 7.61729 | 1 | 0.00578113 | ω0 = 0.03365= 1.00000ω2 =19.37846 | 661 I 0.989*  754 S 0.969* |
|  |  | Ma0 | -24126.161904 |  |  |  |  | ω0 = 0.03339ω1 = 1.00000ω2 = 1.00000 |  |
| *GALNS* | *Eurypyga helias* | Ma | -10844.962113 | Ma vs Ma0 | 11.255788 | 1 | 0.000793751 | ω0 = 0.03770= 1.00000ω2 = 480.10198 | 184 Q 0.890  263 S 0.992** |
|  |  | Ma0 | -10850.590007 |  |  |  |  | ω0 = 0.03736= 1.00000ω2 = 1.00000 |  |
| *GALNS* | *Aquila chrysaetos* | Ma | -10835.837898 | Ma vs Ma0 | 28.574352 | 1 | 9.01686E-08 | ω0 = 0.03858= 1.00000ω2 = 946.12794 | 482 - 0.997**  483 - 0.999** |
|  |  | Ma0 | -10850.125074 |  |  |  |  | ω0 = 0.03855ω1 = 1.00000ω2 = 1.00000 |  |
| *GRB10* | *Melospiza melodia* | Ma | -7875.835243 | Ma vs Ma0 | 15.384102 | 1 | 8.77233E-05 | ω0 = 0.01367ω1 = 1.00000ω2 = 501.92037 | 594 V 0.998** |
|  |  | Ma0 | -7883.527294 |  |  |  |  | ω0 = 0.01366ω1 = 1.00000ω2 = 1.00000 |  |
| *GRB10* | *Patagioenas fasciata* | Ma | -7878.858098 | Ma vs Ma0 | 13.815324 | 1 | 0.000201685 | ω0 = 0.01376= 1.00000ω2 = 730.61546 | 93 P 0.962* |
|  |  | Ma0 | -7885.765760 |  |  |  |  | ω0 = 0.01376ω1 = 1.00000ω2 = 1.00000 |  |
| *IGFBP7* | *Nipponia nippon* | Ma | -4383.305047 | Ma vs Ma0 | 6.140898 | 1 | 0.013209022 | ω0 = 0.03645ω1 = 1.00000ω2 = 17.91863 | 1 - 0.960*  150 S 0.991** |
|  |  | Ma0 | -4386.375496 |  |  |  |  | ω0 = 0.03648ω1 = 1.00000ω2 = 1.00000 |  |
| *IGFBP7* | *Anas platyrhynchos* | Ma | -4385.612126 | Ma vs Ma0 | 7.290692 | 1 | 0.006931277 | ω0 = 0.03708= 1.00000ω2 =321.27887 | 9 - 0.926  98 P 0.726 |
|  |  | Ma0 | -4389.257472 |  |  |  |  | ω0 = 0.03763ω1 = 1.00000ω2 = 1.00000 |  |
| *IGFBP7* | *Phasianus colchicus* | Ma | -4368.931729 | Ma vs Ma0 | 22.615688 | 1 | 1.97862E-06 | ω0 = 0.03120ω1 = 1.00000ω2 = 422.81208 | 275 D 0.998**  278 A 0.857  279 E 0.999**  280 L 0.852 |
|  |  | Ma0 | -4380.239573 |  |  |  |  | ω0 = 0.03139ω1 = 1.00000ω2 = 1.00000 |  |
| *NCAPG* | *Corvus hawaiiensis* | Ma | AAAAAAA | Ma vs Ma0 | 4.96947 | 1 | 0.025798552 | ω0 = 0.03811ω1 = 1.00000ω2 =28.65334 | 75 A 0.972* |
|  |  | Ma0 | -15804.003798 |  |  |  |  | ω0 = 0.03806ω1 = 1.00000ω2 =1.00000 |  |
| *NCAPG* | *Struthio camelus* | Ma | -33679.037275 | Ma vs Ma0 | 6.414506 | 1 | 0.011319181 | ω0 = 0.11885ω1= 1.00000ω2 = 38.39207 | 424 H 0.879 |
|  |  | Ma0 | -33682.244528 |  |  |  |  | ω0 = 0.11851ω1 = 1.00000ω2 = 1.00000 |  |
| *NCAPG* | *Tinamus guttatus* | Ma | -33672.229292 | Ma vs Ma0 | 11.71181 | 1 | 0.000621047 | ω0 = 0.11779ω1 = 1.00000ω2 = 601.92300 | 331 E 0.940  678 N 0.897  1029 S 0.982* |
|  |  | Ma0 | -33678.085197 |  |  |  |  | ω0 = 0.11577ω1 = 1.00000ω2 = 1.00000 |  |
| *NCAPG* | *Tyto alba* | Ma | -33667.005601 | Ma vs Ma0 | 5.628352 | 1 | 0.017672238 | ω0 = 0.11461ω1 = 1.00000ω2 = 3.14712 | 518 S 0.976*  573 I 0.809  605 M 0.906 |
|  |  | Ma0 | -33669.819777 |  |  |  |  | ω0 = 0.11440ω1 = 1.00000ω2 = 1.00000 |  |
| *NCAPG* | *Aptenodytes forsteri* | Ma | -33675.501729 | Ma vs Ma0 | 11.363904 | 1 | 0.000748853 | ω0 = 0.11876ω1 = 1.00000ω2 = 802.67200 | 212 L 0.967* |
|  |  | Ma0 | -33681.844154 |  |  |  |  | ω0 = 0.11879ω1 = 1.00000ω2 = 1.00000 |  |
| *OBSL1* | *Neopelma chrysocephalum* | Ma | -67004.739737 | Ma vs Ma0 | 9.444796 | 1 | 0.002117491 | ω0 = 0.06714ω1 = 1.00000ω2 = 58.25026 | 811 T 0.644  910 S 0.618  2119 G 0.838 |
|  |  | Ma0 | -67009.462135 |  |  |  |  | ω0 = 0.06702ω1 = 1.00000ω2 =1.00000 |  |
| *OBSL1* | *Falco rusticolus* | Ma | -66979.763915 | Ma vs Ma0 | 16.392664 | 1 | 5.14841E-05 | ω0 = 0.06686= 1.00000ω2 = 999.00000 | 526 V 0.919  562 P 0.920  587 G 0.904  595 S 0.921  597 P 0.921  598 R 0.899  599 E 0.999**  643 D 0.988*  720 A 0.921  895 V 0.917 |
|  |  | Ma0 | -67001.498404 |  |  |  |  | ω0 = 0.06685ω1 = 1.00000ω2 = 1.00000 |  |
| *OBSL1* | *Egretta garzetta* | Ma | -66992.389658 | Ma vs Ma0 | 30.767578 | 1 | 2.90857E-08 | ω0 = 0.06686ω1 = 1.00000ω2 = 126.07981 | 340 S 0.990*  347 K 0.983*  1147 R 0.977* |
|  |  | Ma0 | -67007.773447 |  |  |  |  | ω0 = 0.06676ω1 = 1.00000ω2 = 1.00000 |  |
| *OBSL1* | *Pygoscelis adeliae* | Ma | -66987.904008 | Ma vs Ma0 | 20.937294 | 1 | 4.6247E-o8 | ω0 = 0.06685ω1 = 1.00000ω2 = 197.01131 | 1070 D 0.993**  1074 S 0.863  1076 H 0.997**  1170 K 0.824  1733 L 0.839  1737 T 0.849  1743 D 0.999**  1885 Y 0.848  1901 E 0.822  1902 P 0.813  2051 Q 0.872  2056 E 0.838 |
|  |  | Ma0 | -67002.872655 |  |  |  |  | ω0 = 0.06680ω1 = 1.00000ω2 = 1.00000 |  |
| *OBSL1* | *Pygoscelis papua* | Ma | -66998.668163 | Ma vs Ma0 | 22.928222 | 1 | 1.68165E-06 | ω0 = 0.06710ω1 = 1.00000ω2 = 999.00000 | 2025 K 0.987*  2028 K 0.947 |
|  |  | Ma0 | -67010.132274 |  |  |  |  | ω0 = 0.06710ω1 = 1.00000ω2 = 1.00000 |  |
| *OBSL1* | *Mesitornis unicolor* | Ma | -67006.113109 | Ma vs Ma0 | 4.502846 | 1 | 0.03383849 | ω0 = 0.06683ω1 = 1.00000ω2 = 16.19472 | 160 S 0.983*  652 E 0.970*  1819 V 0.835 |
|  |  | Ma0 | -67008.364532 |  |  |  |  | ω0 =ω1 =ω2 = |  |
| *OBSL1* | *Anas platyrhynchos* | Ma | -66999.851323 | Ma vs Ma0 | 19.652844 | 1 | 9.2865E-06 | ω0 = 0.06691ω1 = 1.00000ω2 = 999.00000 | 142 C 0.984*  1463 Q 0.945 |
|  |  | Ma0 | -67009.677745 |  |  |  |  | ω0 = 0.06698ω1 =1.00000ω2 =1.00000 |  |
| *OBSL1* | *Tinamus guttatus* | Ma | -66999.470708 | Ma vs Ma0 | 18.541098 | 1 | 1.6628E-05 | ω0 =0.06692 ω1=1.00000 ω2 =197.79663 | 840 L 0.982*  850 Y 0.978* |
|  |  | Ma0 | -67008.741257 |  |  |  |  | ω0 =0.06674 ω1=1.00000 ω2 =1.00000 |  |
| *OBSL1* | *Dromaius novaehollandiae* | Ma | -66988.972390 | Ma vs Ma0 | 18.186648 | 1 | 2.00278E-05 | ω0 =0.06692 ω1=1.00000 ω2 =197.79663 | 704 T 0.987*  831 G 0.987*  922 G 0.884  1009 G 0.985*  1611 A 0.906  1745 T 0.987*  1951 R 0.806 |
|  |  | Ma0 | -66998.065714 |  |  |  |  | ω0 =0.06674 ω1=1.00000 ω2 =1.00000 |  |
| *OBSL1* | *Struthio camelus* | Ma | -67002.892812 | Ma vs Ma0 | 7.244928 | 1 | 0.007110157 | ω0 =0.06679 ω1=1.00000 ω2 =16.66110 | 778 C 0.982*  1122 D 0.985* |
|  |  | Ma0 | -67006.515276 |  |  |  |  | ω0 =0.06671 ω1=1.00000 ω2 =1.00000 |  |
| *PLAG1* | *Hirundo rustica* | Ma | -15801.519063 | Ma vs Ma0 | 4.96947 | 1 | 0.025798552 | ω0 = 0.03811ω1 = 1.00000ω2 = 28.65334 | 72 A 0.972* |
|  |  | Ma0 | -15804.003798 |  |  |  |  | ω0 = 0.03806ω1 = 1.00000ω2 = 1.00000 |  |
| *PLOD1* | *Hirundo rustica* | Ma | -15801.519063 | Ma vs Ma0 | 4.96947 | 1 | 0.025798552 | ω0 = 0.03811ω1 = 1.00000ω2 = 28.65334 | 75 A 0.972* |
|  |  | Ma0 | -15804.003798 |  |  |  |  | ω0 = 0.03806ω1 = 1.00000ω2 = 1.00000 |  |
| *IGF2BP1* | *Sturnus vulgaris* | Ma | 15140.326691 | Ma vs Ma0 | 122.881038 | 1 | 1.48066E-28 | ω0=0.04797  ω1=1.00000  ω2=905.58810 | 32 Q 0.909  150 K 1.000**  151 V 0.975*  153 Y 1.000**  176 A 0.927  178 R 1.000**  179 Q 0.878  181 S 1.000**  196 I 0.971*  197 P 1.000**  199 R 1.000**  200 L 0.933  201 L 0.963*  202 V 0.945  204 T 1.000**  205 Q 0.969*  207 V 0.942  208 G 1.000**  209 A 0.962*  210 I 0.972*  211 I 0.963*  234 K 0.970*  235 E 1.000**  236 N 1.000**  238 G 1.000**  239 A 1.000**  240 A 0.961*  242 K 1.000**  243 A 0.957*  244 I 0.999**  245 S 1.000**  246 I 0.972*  247 H 0.942  248 S 1.000**  249 T 0.968*  251 E 0.961*  303 E 0.999**  304 Q 1.000**  306 T 0.993**  309 K 1.000**  310 I 0.987*  311 T 1.000**  317 D 0.969*  318 L 0.970*  320 L 1.000**  321 Y 1.000**  323 P 0.955*  325 R 0.944  332 S 0.978*  346 K 0.961*  383 V 0.942  388 S 0.902  547 M 0.894  548 A 1.000** |
|  |  | Ma0 | 15201.76721 |  |  |  |  | ω0= 0.04761  ω1=1.00000  ω2=1.00000 |  |
| *IGF2BP1* | *Cyanistes caeruleus* | Ma | 15151.758697 | Ma vs Ma0 | 114.49385 | 1 | 1.01581E-26 | ω0=0.05022  ω1=1.00000  ω2=666.63240 | 24 D 0.999**  25 H 0.999**  26 K 0.944  27 I 1.000**  28 S 1.000**  29 F 0.941  30 S 0.970*  31 G 1.000**  33 F 0.941  40 A 0.938  41 F 0.836  42 V 1.000**  43 D 0.987*  44 C 0.987*  74 V 1.000**  76 K 1.000**  77 K 1.000**  78 Q 0.998**  79 R 0.982*  80 S 0.925  81 R 1.000**  82 K 1.000**  84 Q 0.989*  85 I 0.891  86 R 0.924  87 N 0.999**  89 P 0.997**  90 P 0.933  91 Q 1.000**  92 L 0.961*  93 R 1.000**  140 N 0.999**  169 G 0.930  170 G 0.908  172 G 0.976*  175 G 0.910  176 A 0.921  177 P 0.973*  180 G 1.000**  182 P 0.925  185 A 0.997**  186 G 0.994**  188 P 0.999**  189 V 0.973*  192 Q 0.924  193 P 1.000**  194 V 0.974*  195 D 0.999**  198 L 0.957*  199 R 0.977*  200 L 0.952*  201 L 0.927  204 T 1.000**  206 Y 0.881  207 V 0.913  210 I 0.955*  211 I 0.998**  213 K 1.000**  214 E 1.000**  216 A 1.000**  217 T 0.812  226 Q 0.986*  227 S 0.857 |
|  |  | Ma0 | 15209.005622 |  |  |  |  | ω0=0.04906  ω1= 1.00000  ω2=1.00000 |  |
| *IGF2BP1* | *Pseudopodoces humilis* | Ma | 15253.947107 | Ma vs Ma0 | 39.97961 | 1 | 2.56628E-10 | ω0 = 0.05395  ω1= 1.00000  ω2=999.00000 | 136 I 0.986*  137 M 0.801  138 K 0.921  150 K 0.999**  169 G 0.947  194 V 0.868 |
|  |  | Ma0 | 15273.936912 |  |  |  |  | ω0=0.05394  ω1=1.000  ω2=1.000 |  |
| *IGF2BP1* | *Egretta garzetta* | Ma | 15193.796924 | Ma vs Ma0 | 100.772724 | 1 | 1.03167E-23 | ω0=0.05209  ω1=1.000  ω2=699.97199 | 21 V 0.924  22 F 0.999**  25 H 1.000**  26 K 0.949  39 Y 0.959*  40 A 0.820  41 F 0.935  42 V 0.999**  43 D 0.999**  48 Q 0.919  49 W 0.953*  50 A 0.999**  51 M 0.922  53 A 0.995**  54 I 0.997**  55 E 0.952*  56 T 0.903  58 S 0.858  158 Q 0.896  196 I 0.934  199 R 1.000**  202 V 0.834  203 P 0.893  205 Q 0.998**  206 Y 0.922  209 A 0.999**  210 I 0.972*  216 A 1.000**  218 I 0.998**  221 I 0.934  222 T 1.000**  224 Q 0.998**  227 S 0.910  230 D 0.999**  231 V 0.933  272 K 0.999**  273 T 0.941 |
|  |  | Ma0 | 15244.183286 |  |  |  |  | ω0=0.05113  ω1=1.000  ω2=1.000 |  |
| *IGF2BP1* | *Pygoscelis adeliae* | Ma | 15253.607415 | Ma vs Ma0 | 40.64229 | 1 | 1.82807E-10 | ω0=0.05457  ω1 =1.000  ω2=906.85289 | 45 P 1.000**  46 D 0.999**  134 Q 0.985*  187 A 0.963*  188 P 0.908  441 A 0.964* |
|  |  | Ma0 | 15273.928560 |  |  |  |  | ω0=0.05440  ω1=1.000  ω2=1.000 |  |
| *IGF2BP1* | *Megadyptes antipodes* | Ma | 15275.330779 | Ma vs Ma0 | 8.501242 | 1 | 0.003549041 | ω0=0.05495  ω1=1.000  ω2=559.62871 | 134 Q 0.874 |
|  |  | Ma0 | 15279.581400 |  |  |  |  | ω0=0.05489  ω1=1.000  ω2=1.000 |  |
| *IGF2BP1* | *Eudyptes pachyrhynchus* | Ma | 15274.286334 | Ma vs Ma0 | 10.363746 | 1 | 0.001285142 | ω0=0.05495  ω1=1.000  ω2=999.00000 | 134 Q 0.867 |
|  |  | Ma0 | 15279.468207 |  |  |  |  | ω0=0.05491  ω1=1.000  ω2=1.000 |  |
| *IGF2BP1* | *Meleagris gallopavo* | Ma | 15194.460358 | Ma vs Ma0 | 126.636328 | 1 | 2.23135E-29 | ω0=0.05372  ω1=1.000  ω2=999.00000 | 15 P 1.000**  16 A 1.000**  17 D 0.938  18 L 1.000**  19 E 1.000**  20 K 0.994**  21 V 1.000**  22 F 0.945  23 N 0.927  46 D 1.000**  47 E 0.899  49 W 0.991**  50 A 0.999**  51 M 1.000**  53 A 0.986*  54 I 0.999**  55 E 0.987*  56 T 0.971*  58 S 0.939 |
|  |  | Ma0 | 15257.778522 |  |  |  |  | ω0=0.05316  ω1=1.000  ω2=1.000 |  |
| *IGF2BP1* | *Tinamus guttatus* | Ma | 15248.319452 | Ma vs Ma0 | 45.2223 | 1 | 1.75891E-11 | ω0= 0.05441  ω1=1.000  ω2=507.33773 | 33 F 0.986*  38 G 0.922  40 A 0.986*  41 F 0.868  44 C 0.855  46 D 0.964*  50 A 0.996**  51 M 0.991**  52 K 0.894  53 A 0.854  54 I 0.994**  57 F 0.999** |
|  |  | Ma0 | 15270.930602 |  |  |  |  | ω0=0.05374  ω1= 1.00000  ω2=1.00000 |  |
| *IGF2BP1* | *Struthio camelus* | Ma | 15055.896252 | Ma vs Ma0 | 214.053484 | 1 | 1.79287E-48 | ω0=0.04612  ω1=1.000  ω2=873.82116 | 12 N 0.882  13 V 1.000**  14 T 0.975*  17 D 0.991**  18 L 0.951*  19 E 0.899  20 K 0.998**  21 V 0.986*  22 F 1.000**  23 N 0.874  25 H 0.993**  27 I 0.999**  29 F 0.897  30 S 1.000**  31 G 1.000**  33 F 0.992**  34 L 1.000**  35 V 0.950*  36 K 1.000**  37 S 0.880  38 G 0.915  39 Y 0.989*  40 A 0.886  42 V 0.969*  43 D 0.999**  44 C 0.924  46 D 0.999**  47 E 0.990**  49 W 0.860  50 A 0.848  51 M 0.981*  52 K 0.988*  53 A 0.961*  54 I 0.992**  55 E 0.974*  57 F 1.000**  58 S 0.999**  59 G 0.841  60 K 0.999**  62 E 0.950*  64 H 1.000**  69 E 1.000**  70 I 1.000**  71 E 1.000**  72 H 1.000**  73 S 0.963*  74 V 1.000**  75 P 1.000**  76 K 0.999**  77 K 0.994**  78 Q 1.000**  203 P 0.906  205 Q 0.899  440 I 0.977*  442 P 1.000**  443 P 0.933  445 T 1.000**  447 D 0.988*  448 S 0.952*  449 K 1.000**  450 V 0.996**  451 R 0.965*  452 M 1.000**  453 V 0.959*  454 V 0.965*  455 I 0.993**  456 T 0.926  459 P 1.000**  460 E 0.960*  461 A 0.952*  462 Q 0.933  463 F 0.954*  464 K 0.899  517 A 0.972*  518 A 0.909  519 E 1.000**  520 V 1.000**  521 V 0.977*  522 V 1.000**  525 D 1.000**  526 Q 0.993**  528 P 1.000**  529 D 1.000**  530 E 1.000**  531 N 0.988*  532 E 1.000**  533 Q 1.000**  534 V 1.000**  535 I 0.956*  536 V 0.977*  539 I 0.816  540 G 1.000**  541 H 0.939  543 Y 1.000**  544 A 1.000**  545 S 0.987*  546 Q 0.899 |
|  |  | Ma0 | 15162.922994 |  |  |  |  | ω0=0.05374  ω1= 1.00000  ω2=1.00000 |  |
| *IGF2BP1* | *Parus major* | Ma | 15123.112486 | Ma vs Ma0 | 130.819476 | 1 | 2.7118E-30 | ω0=0.04716  ω1=1.000  ω2=999.00000 | 136 I 0.997**  137 M 1.000**  138 K 1.000**  139 L 0.960*  140 N 0.971*  150 K 1.000**  151 V 0.966*  152 S 1.000**  153 Y 1.000**  154 I 0.965*  195 D 1.000**  274 A 1.000**  275 D 0.971*  276 E 0.956*  345 K 0.944  347 V 0.993**  349 E 0.934  350 A 1.000**  351 Y 1.000**  352 E 0.959*  353 N 1.000**  445 T 0.966*  518 A 0.957*  519 E 1.000**  520 V 0.986*  521 V 0.969*  522 V 0.993**  523 P 1.000**  524 R 1.000**  525 D 0.995**  526 Q 1.000**  529 D 1.000**  530 E 0.997**  531 N 0.997**  533 Q 0.961*  534 V 1.000**  535 I 0.962*  536 V 0.975*  537 K 1.000**  538 I 1.000**  539 I 0.996**  540 G 0.995**  541 H 0.955*  543 Y 0.964*  545 S 0.947  546 Q 1.000**  547 M 0.994**  548 A 0.972*  549 Q 1.000**  551 K 1.000**  552 I 0.962*  555 I 1.000**  557 A 0.966*  567 Q 1.000**  568 S 1.000**  569 S 0.806  570 Q 0.860  573 A 0.966*  574 R 1.000**  575 R 1.000**  576 K 1.000** |
|  |  | Ma0 | 15188.522224 |  |  |  |  | ω0=0.04671  ω1= 1.00000  ω2=1.00000 |  |
| *ATP11A* | *Callipepla squamata* | Ma | 20483.32255 | Ma vs Ma0 | 152.934042 | 1 | 3.95993E-35 | ω0=0.03118 ω1=1.00000 ω2=728.01004 | 12 R 0.984*  13 Y 0.906  265 V 0.991**  266 A 0.995**  267 I 0.817  268 Y 0.984*  269 T 0.954*  270 G 0.805  271 M 0.991**  272 E 0.998**  273 T 0.806  274 K 0.809  276 A 0.935  277 L 0.968*  278 N 0.816  280 Q 0.984*  283 S 0.991**  289 V 0.996**  290 E 0.991** |
|  |  | Ma0 | 20559.78957 |  |  |  |  | ω0=0.03134 ω1=1.00000 ω2=1.00000 |  |
| *ATP11A* | *Corvus kubaryi* | Ma | 20572.34184 | Ma vs Ma0 | 6.016782 | 1 | 0.014170462 | ω0=0.03381 ω1=1.00000 ω2=12.46915 | 815 K 0.961* |
|  |  | Ma0 | 20575.35023 |  |  |  |  | ω0=0.03375 ω1=1.00000 ω2=1.00000 |  |
| *PLXDC2* | *Aptenodytes patagonicus* | Ma | 10172.95634 | Ma vs Ma0 | 20812.47296 | 8 | 0 | ω0=0.05310 ω1=1.00000 ω2=3.01737 | 491 E 0.893 |
|  |  | Ma0 | 20579.19282 |  |  |  |  | ω0=0.03451 ω1=1.00000 ω2=1.00000 |  |
| *ATP11A* | *Nipponia nippon* | Ma | 20576.39946 | Ma vs Ma0 | 5.122616 | 1 | 0.02361599 | ω0=0.03494 ω1=1.00000 ω2=30.37402 | 1126 H 0.932 |
|  |  | Ma0 | 20578.96076 |  |  |  |  | ω0=0.03490 ω1=1.00000 ω2=1.00000 |  |
| *TUBGCP3* | *Melospiza melodia* | Ma | 17743.97304 | Ma vs Ma0 | 3.861642 | 1 | 0.049401958 | ω0=0.01865 ω1=1.00000 ω2=19.18729 | 866 T 0.899 |
|  |  | Ma0 | 17745.90386 |  |  |  |  | ω0=0.01868 ω1=1.00000 ω2=1.00000 |  |
| *TUBGCP3* | *Corvus kubaryi* | Ma | 17673.377 | Ma vs Ma0 | 79.728468 | 1 | 4.29567E-19 | ω0=0.01682 ω1=1.00000 ω2=999.00000 | 882 F 0.947  883 N 1.000**  84 E 1.000**  885 H 1.000**  886 Y 1.000**  887 K 1.000**  888 A 0.945 |
|  |  | Ma0 | 17713.24124 |  |  |  |  | ω0=0.01868 ω1=1.00000 ω2=1.00000 ω1=1.00000 ω2=1.00000 |  |
| *TNS3* | *Zonotrichia albicollis* | Ma | 43032.21117 | Ma vs Ma0 | 115.720832 | 1 | 5.47142E-27 | ω0=0.12124 ω1=1.00000 ω2=999.00000 | 101 E 0.805 |
|  |  | Ma0 | 43090.07158 |  |  |  |  | ω0=0.12109 ω1=1.00000 ω2=1.00000 |  |
| *TNS3* | *Lonchura striata* | Ma | 43078.313195 | Ma vs Ma0 | 4.30673 | 1 | 0.037961866 | ω0=0.11794 ω1=1.00000 ω2=4.13719 | 503 P 0.964*  1442 A 0.960*  1492 V 0.915 |
|  |  | Ma0 | 43080.466560 |  |  |  |  | ω0= 0.11759 ω1=1.00000 ω2=1.00000 |  |
| *TNS3* | *Corvus kubaryi* | Ma | 42774.94516 | Ma vs Ma0 | 471.782306 | 1 | 1.312E-104 | ω0=0.11243 ω1=1.00000 ω2=999.00000 | 547 G 0.964*  599 V 0.801  814 R 0.950*  965 E 0.999**  966 I 0.928  967 D 0.996**  968 G 1.000**  974 A 0.884  975 K 1.000**  977 G 0.999**  978 N 0.950*  979 E 1.000**  980 S 0.997**  981 V 0.986*  982 P 0.997**  983 S 0.856  984 T 0.998**  985 P 0.992**  986 G 0.982*  988 P 0.831  1001 L 0.913  1081 S 0.971*  1082 S 0.969*  1084 T 0.994**  1085 K 0.846  1086 E 0.959*  1087 C 0.894  1088 H 0.870  1090 L 0.869  1091 E 1.000**  1092 S 0.986*  1093 S 0.971*  1095 K 0.959*  1096 S 1.000**  1098 P 0.851  1099 S 0.969*  1100 L 0.851  1101 Q 0.998**  1127 S 1.000**  1129 L 0.978*  1130 S 0.959*  1131 H 0.992**  1132 F 0.968*  1133 Q 0.964*  1134 T 0.967*  1135 P 0.999**  1182 K 0.998**  1183 Q 0.999**  1184 A 0.869  1185 T 0.842  1186 F 0.858  1187 I 0.997**  1189 N 0.998**  1190 T 0.907  1191 S 0.801  1192 I 0.981*  1193 S 0.995**  1194 P 0.999**  1195 K 0.963*  1196 T 0.986*  1197 M 0.879  1198 S 0.999**  1199 S 0.932  1200 S 0.907  1201 V 0.991**  1203 S 0.988*  1204 A 0.969*  1229 H 0.975*  1230 Q 0.890  1231 H 0.996**  1232 L 0.987*  1235 N 0.982*  1236 L 0.620  1253 R 0.982*  1254 S 1.000**  1255 F 0.926  1256 T 0.965*  1257 S 0.971*  1258 V 0.990*  1259 S 0.996**  1260 P 1.000**  1262 S 1.000**  1263 S 0.995**  1265 F 1.000**  1266 S 0.999**  1380 H 0.972*  1382 L 0.999**  1383 I 0.972*  1384 E 0.999**  1386 T 1.000**  1387 Q 0.925  1388 K 1.000**  1390 V 0.965*  1391 R 0.999**  1392 L 0.991**  1393 K 1.000**  1396 P 0.966*  1513 F 1.000**  1514 R 0.999**  1515 R 0.969*  1516 H 1.000**  1517 Y 0.970*  1518 P 1.000**  1519 A 1.000**  1520 N 0.911  1521 T 0.999**  1522 V 1.000**  1523 I 0.968*  1524 F 0.973*  1546 F 0.968*  1547 V 0.970*  1548 A 0.982*  1549 R 0.998**  1551 Q 0.999**  1552 G 0.999**  1553 S 0.970*  1554 A 0.999**  1555 T 0.999**  1556 D 0.996**  1557 N 0.999**  1558 V 0.987*  1561 L 1.000**  1562 F 1.000**  1563 A 1.000**  1564 E 1.000**  1565 H 0.993**  1566 D 1.000**  1567 P 0.964*  1568 E 1.000**  1569 Q 0.991**  1570 P 0.952*  1571 A 0.971*  1572 S 0.958*  1573 A 0.956*  1574 I 0.962*  1575 V 1.000**  1576 N 0.999**  1577 F 0.973*  1578 V 1.000**  1579 S 0.974*  1580 K 0.965*  1581 V 0.968*  1582 M 1.000**  1584 G 0.997**  1585 S 0.999**  1586 Q 0.861  1587 K 0.995**  1588 K 0.998** |
|  |  | Ma0 | 43010.836312 |  |  |  |  | ω0=0.11110 ω1=1.00000 ω2=1.00000 |  |
| *TNS3* | *Hirundo rustica* | Ma | 42959.449354 | Ma vs Ma0 | 155.252712 | 1 | 1.23302E-35 | ω0=0.11766 ω1=1.00000 ω2=999.00000 | 62 D 0.990**  64 R 0.864  65 G 0.993**  66 I 0.806  592 I 0.936  707 P 0.970*  959 V 0.806  1161 S 0.970*  1543 V 0.995**  1545 G 0.999**  1550 K 1.000**  1551 Q 0.982*  1552 G 0.899  1553 S 0.995**  1555 T 0.905  1556 D 0.999**  1557 N 0.980*  1560 H 0.994**  1565 H 0.999**  1568 E 0.903  1569 Q 0.996**  1570 P 0.999**  1571 A 0.807  1572 S 0.988*  1573 A 0.989*  1574 I 0.918  1575 V 0.820  1576 N 0.988*  1578 V 0.962*  1580 K 0.977*  1583 I 0.988*  1584 G 0.983*  1586 Q 0.964*  1587 K 0.999**  1588 K 0.997** |
|  |  | Ma0 | 43037.075710 |  |  |  |  | ω0=0.11453 ω1=1.00000 ω2=1.00000 |  |
| *TNS3* | *Charadrius vociferu* | Ma | 43071.220829 | Ma vs Ma0 | 36.502552 | 1 | 1.52467E-09 | ω0=0.12147 ω1=1.00000 ω2=863.95406 | 903 D 0.938  1219 S 0.992**  1233 G 0.954*  1234 G 0.957* |
|  |  | Ma0 | 43089.472105 |  |  |  |  | ω0= 0.12108  ω1=1.00000 ω2=1.00000 |  |
| *TNS3* | *Egretta garzetta* | Ma | 43078.025081 | Ma vs Ma0 | 24.11969 | 1 | 9.05299E-07 | ω0= 0.12111 ω1=1.00000 ω2=74.93029 | 49 K 0.894  52 K 0.873  69 R 0.807  780 L 0.862  864 N 0.981*  894 S 0.888  1177 S 0.869 |
|  |  | Ma0 | 43090.084926 |  |  |  |  | ω0=0.12090 ω1=1.00000 ω2=1.00000 |  |
| *TNS3* | *Pygoscelis papua* | Ma | 43003.972935 | Ma vs Ma0 | 134.758394 | 1 | 3.72892E-31 | ω0=0.11842 ω1=1.00000 ω2=999.00000 | 114 S 0.950  123 C 0.827  124 D 0.943  203 I 0.998**  204 M 0.901  206 V 0.894  207 G 0.998**  208 W 0.997**  235 Q 0.899  236 H 0.801  237 V 0.907  239 V 1.000**  240 I 0.905  241 H 0.998**  351 Y 0.883  1369 K 0.886  1542 K 0.900 |
|  |  | Ma0 | 43071.352132 |  |  |  |  | ω0=0.11813 ω1=1.00000 ω2=1.00000 |  |
| *TNS3* | *Aptenodytes patagonicus* | Ma | 43084.810569 | Ma vs Ma0 | 8.27513 | 1 | 0.004019178 | ω0= 0.12073 ω1=1.00000 ω2=278.16809 | 265 S 0.973* |
|  |  | Ma0 | 43088.948134 |  |  |  |  | ω0=0.12077 ω1=1.00000 ω2=1.00000 |  |
| *TNS3* | *Meleagris gallopavo* | Ma | 43068.756506 | Ma vs Ma0 | 40.394316 | 1 | 2.07544E-10 | ω0=0.12147 ω1=1.00000 ω2=999.00000 | 378 K 0.975*  381 H 0.975*  382 K 0.995** |
|  |  | Ma0 | 43088.953664 |  |  |  |  | ω0=0.12108 ω1=1.00000 ω2=1.00000 |  |
| *TNS3* | *Tinamus guttatus* | Ma | 43078.729938 | Ma vs Ma0 | 17.961094 | 1 | 2.25466E-05 | ω0=0.12065 ω1=1.00000 ω2=108.00233 | 242 C 0.964*  296 Q 0.937  297 F 0.952*  351 Y 0.939 |
|  |  | Ma0 | 43087.710485 |  |  |  |  | ω0=0.12000 ω1=1.00000 ω2=1.00000 |  |
| *ATP11A* | *Aptenodytes forsteri* | Ma | 20564.875865 | Ma vs Ma0 | 29.60675 | 1 | 5.29203E-08 | ω0=0.03492  ω1=1.00000  ω2=999.00000 | 807 I 0.933 |
|  |  | Ma0 | 20579.679240 |  |  |  |  | ω0=0.03495  ω1=1.00000  ω2=1.00000 |  |
| *PLXDC2* | *Charadrius vociferus* | Ma | 10149.746431 | Ma vs Ma0 | 49.5084 | 1 | 1.97524E-12 | ω0=0.05692  ω1=1.00000  ω2=239.91971 | 5 K 0.936  7 - 0.971*  14 - 0.856  18 - 0.992** |
|  |  | Ma0 | 10174.500631 | Ma vs Ma0 |  |  |  | ω0=0.05361  ω1=1.00000  ω2=1.00000 |  |
| *PLXDC2* | *Anas platyrhynchos* | Ma | 10084.474253 | Ma vs Ma0 | 160.416364 | 1 | 9.17653E-37 | ω0=0.05820  ω1=1.00000  ω2=999.00000 | 1 M 1.000**  3 K 0.886  8 - 1.000**  9 - 0.989*  10 - 0.933  11 - 0.994**  12 - 0.960*  13 - 0.999**  14 - 0.814  15 - 0.985*  16 - 0.938  17 - 0.959*  19 - 0.991**  20 - 0.998**  21 - 0.927  22 - 1.000**  23 - 0.957*  25 - 0.988*  27 L 0.929  28 G 0.900  29 D 0.885  30 E 0.930  31 V 0.984*  32 - 0.927  34 - 0.986* |
|  |  | Ma0 | 10164.682435 |  |  |  |  | ω0=0.05316  ω1=1.00000  ω2=1.00000 |  |
| *PLXDC2* | *Oxyura jamaicensis* | Ma | 10170.610748 | Ma vs Ma0 | 7.682898 | 1 | 0.005574657 | ω0=0.05344  ω1=1.00000  ω2=616.63409 | 3 K 0.818 |
|  |  | Ma0 | 10174.452197 |  |  |  |  | ω0=0.05320  ω1=1.00000  ω2=1.00000 |  |
| *PLXDC2* | *Meleagris gallopavo* | Ma | 10031.420168 | Ma vs Ma0 | 120.22105 | 1 | 5.65901E-28 | ω0=0.03104  ω1=1.00000  ω2=999.00000 | 124 D 0.908  125 S 1.000**  128 R 0.996**  158 V 0.989*  159 N 0.999**  206 D 1.000**  208 S 1.000**  209 V 0.948  210 S 1.000**  211 R 0.918  212 N 1.000**  213 S 0.949  234 Q 0.955*  235 D 1.000**  237 Y 1.000**  238 N 1.000**  239 L 1.000**  240 G 1.000**  241 S 1.000**  242 F 0.946  243 T 0.994**  244 F 0.941  245 Q 0.940  246 A 1.000**  247 T 1.000**  252 G 0.934  253 R 1.000**  254 I 1.000**  255 I 1.000**  257 G 0.999**  258 Y 0.999**  259 K 0.931  260 E 1.000**  332 S 0.904 |
|  |  | Ma0 | 10091.530693 |  |  |  |  | ω0=0.05320  ω1=1.00000  ω2=1.00000 |  |
| *PLXDC2* | *Strigops habroptila* | Ma | 10165.768285 | Ma vs Ma0 | 12.955858 | 1 | 0.000318922 | ω0=0.05132  ω1=1.00000  ω2=998.99995 | 439 S 0.993**  524 I 0.812 |
|  |  | Ma0 | 10172.246214 |  |  |  |  | ω0=0.05152  ω1=1.00000  ω2=1.00000 |  |
| *TUBGCP3* | *Sturnus vulgaris* | Ma | 17700.058037 | Ma vs Ma0 | 50.187348 | 1 | 1.39746E-12 | ω0=0.01727  ω1=1.00000  ω2=330.65795 | 74 F 0.998**  75 S 0.972*  76 E 0.998**  79 R 0.997**  81 L 0.999**  83 S 0.999** |
|  |  | Ma0 | 17725.151711 |  |  |  |  | ω0=0.01708  ω1=1.00000  ω2=1.00000 |  |
| *ATP11A* | *Patagioenas fasciata* | Ma | 20545.150663 | Ma vs Ma0 | 56.928624 | 1 | 4.5192E-14 | ω0=0.03459  ω1=1.00000  ω2=186.40803 | 1123 C 0.997**  1155 N 0.994**  1156 S 0.997**  1159 K 0.963*  1160 K 0.946  1186 R 0.834  1189 A 0.997** |
|  |  | Ma0 | 20573.614975 |  |  |  |  | ω0=0.03441  ω1=1.00000  ω2=1.00000 |  |
| *TUBGCP3* | *Struthio camelus* | Ma | 17749.753156 | Ma vs Ma0 | 8.75042 | 1 | 0.003095308 | ω0=0.01893  ω1=1.00000  ω2=1.00000 | 186 Y 0.963* |
|  |  | Ma0 | 17745.377946 |  |  |  |  | ω0=0.01879  ω1=1.00000  ω2=1.00000 |  |
| *ATP11A* | *Tinamus guttatus* | Ma | 20575.432955 | Ma vs Ma0 | 8.291736 | 1 | 0.00398259 | ω0=0.03506  ω1=1.00000  ω2=48.29151 | 53 S 0.947 |
|  |  | Ma0 | 20579.578823 |  |  |  |  | ω0=0.03503  ω1=1.00000  ω2=1.00000 |  |
| *ATP11A* | *Oxyura_jamaicensis* | Ma | 20573.953999 | Ma vs Ma0 | 6.497514 | 1 | 0.010802543 | ω0=0.03437  ω1=1.00000  ω2=67.08980 | 320 S 0.979* |
|  |  | Ma0 | 20577.202756 |  |  |  |  | ω0=0.03428  ω1=1.00000  ω2=1.00000 |  |
| *TUBGCP3* | *Cyanistes_caeruleus* | Ma | 17713.789443 | Ma vs Ma0 | 45.320394 | 1 | 1.67298E-11 | ω0=0.01795  ω1=1.00000  ω2=217.60984 | 15 S 0.906  24 S 0.906  490 I 0.944  493 L 0.997**  494 H 0.998**  495 Q 1.000** |
|  |  | Ma0 | 17736.449640 |  |  |  |  | ω0=0.01793  ω1=1.00000  ω2=1.00000 |  |

**Table S6b** The results of branch-site model for Galliformes.

| **Gene** | **The branch of** | **Model** | **-lnL** | **Model**  **comparison** | **2ΔlnL** | **df** | ***P*-value** | **Parameters** | **Positive site**  **(PP≥80%)** |
| --- | --- | --- | --- | --- | --- | --- | --- | --- | --- |
| *EIF2AK3* | *Meleagris gallopavo* | Ma | 7356.060023 | Ma vs Ma0 | 10.363502 | 1 | 0.001285312 | ω0=0.03268 ω1=1.00000 ω2=653.13267 | 125 P 0.876 |
|  |  | Ma0 | 7361.241774 |  |  |  |  | ω0=0.03119 ω1=1.00000 ω2=1.00000 |  |
| *GALNS* | *Colinus virginianus* | Ma | 3623.172637 | Ma vs Ma0 | 40.03928 | 1 | 2.48907E-10 | ω0=0.04267 ω1=1.00000 ω2=999.00000 | 10 W 0.832  11 L 0.969*  22 N 0.982*  27 L 0.946 |
|  |  | Ma0 | 3643.192277 |  |  |  |  | ω0=0.03854 ω1=1.00000 ω2=1.00000 |  |
| *GALNS* | *Meleagris gallopavo* | Ma | 3595.599371 | Ma vs Ma0 | 78.164752 | 1 | 9.4795E-19 | ω0=0.02358 ω1=1.00000 ω2=830.66235 | 389 Y 1.000**  391 A 1.000**  392 H 0.977*  393 Y 0.995**  394 W 1.000**  397 S 0.999**  398 N 0.974*  399 S 0.999**  430 P 0.995**  433 F 0.995**  480 C 0.999**  482 K 0.979*  483 A 0.993**  484 V 1.000** |
|  |  | Ma0 | 3634.681747 |  |  |  |  | ω0=0.02738 ω1=1.00000 ω2=1.00000 |  |
| *GHSR* | *Centrocercus urophasianus* | Ma | 2288.599714 | Ma vs Ma0 | 110.129796 | 1 | 9.17801E-26 | ω0=0.02935 ω1=1.00000 ω2=156.31924 | 247 V 0.919  249 V 0.843  250 V 0.991  251 F 0.992  284 Y 0.999**  286 N 0.998**  289 S 0.869  290 F 0.986*  309 K 0.882  311 Y 0.999**  312 R 0.962*  314 A 0.987*  315 A 0.998**  318 L 0.801  319 F 0.994**  320 G 0.930  322 K 0.999**  324 P 0.970*  325 P 0.998**  327 K 0.995**  328 R 0.999**  332 T 0.994**  333 K 0.999**  334 Q 0.999**  335 D 0.985*  338 R 0.998** |
|  |  | Ma0 | 2343.664612 |  |  |  |  | ω0=0.01567  ω1=1.00000 ω2=1.00000 |  |
| *GRB10* | *Callipepla squamata* | Ma | 3282.234229 | Ma vs Ma0 | 74.436342 | 1 | 6.26255E-18 | ω0=0.00000  ω1=1.00000 ω2=999.00000 | 73 N 0.933  344 N 0.935  549 V 0.999**  550 S 1.000**  551 N 0.939  584 T 0.938  585 L 0.999**  586 M 0.991**  587 I 0.948  589 K 0.997**  590 E 0.997** |
|  |  | Ma0 | 3319.452400 |  |  |  |  | ω0=0.00000 ω1=1.00000 ω2=1.00000 |  |
| *IGF2BP1* | *Meleagris gallopavo* | Ma | 3599.296712 | Ma vs Ma0 | 123.810418 | 1 | 9.26896E-29 | ω0=0.00432 ω1=1.00000 ω2=999.00000 | 2 N 0.992**  3 K 0.935  4 L 0.999**  16 A 1.000**  22 F 0.937  23 N 0.999**  30 S 0.999**  31 G 0.967*  33 F 0.937  34 L 0.995**  35 V 1.000**  36 K 0.923  37 S 0.998**  38 G 0.999**  39 Y 0.998**  40 A 1.000**  42 V 0.958* |
|  |  | Ma0 | 3661.201921 |  |  |  |  | ω0=0.00391 ω1=1.00000 ω2=1.00000 |  |
| *IGFBP7* | *Coturnix japonica* | Ma | 2337.404485 | Ma vs Ma0 | 10.33783 | 1 | 0.001303311 | ω0=0.00391 ω1=1.00000 ω2=999.00000 | 318 G 0.911 |
|  |  | Ma0 | 2342.573400 |  |  |  |  | ω0=0.00391 ω1=1.00000 ω2=1.00000 |  |
| *IGFBP7* | *Phasianus colchicus* | Ma | 2333.668455 | Ma vs Ma0 | 17.22816 | 1 | 3.31486E-05 | ω0=0.00000 ω1=1.00000 ω2=202.60653 | 1 - 0.827  5 - 0.964* |
|  |  | Ma0 | 2342.282535 |  |  |  |  | ω0=0.00391 ω1=1.00000 ω2=1.00000 |  |
| *NCAPG* | *Colinus virginianus* | Ma | 8446.992065 | Ma vs Ma0 | 119.947408 | 1 | 3.22012E-24 | ω0=0.09831 ω1=1.00000 ω2=645.78743 | 338 V 0.802  377 I 0.817  972 Q 0.997**  973 R 0.980*  974 K 0.999**  975 A 0.990**  976 A 0.997**  977 V 0.963*  979 T 0.999**  980 R 0.988*  981 S 0.810  983 S 0.929  984 R 0.989*  985 R 0.802  986 K 0.820  987 T 0.977*  988 G 0.785  989 T 0.997** |
|  |  | Ma0 | 8506.965769 |  |  |  |  | ω0=0.090621 ω1=1.00000 ω2=1.00000 |  |
| *OBSL1* | *Phasianus colchicus* | Ma | 17360.728106 | Ma vs Ma0 | 252.997408 | 1 | 5.76757E-57 | ω0=0.04465 ω1=1.00000 ω2=999.00000 | 639 V 0.907  754 - 0.869  768 - 0.998  771 - 0.998**  772 - 0.996**  776 - 0.643  777 - 0.965*  778 - 0.656  784 - 0.979*  785 - 0.998**  786 - 0.983* |
|  |  | Ma0 | 17487.226810 |  |  |  |  | ω0=0.03882 ω1=1.00000 ω2=1.00000 |  |
| *ATP11A* | *Colinus virginianus* | Ma | 7975.254180 | Ma vs Ma0 | 435.435676 | 1 | 1.06618E-96 | ω0=0.00000 ω1=1.00000 ω2=999.00000 | 1 M 0.973*  2 D 0.933  1078 G 0.830  1110 N 1.000**  1111 A 0.969*  1112 S 0.998**  1113 R 0.971*  1114 H 1.000**  1115 C 0.996**  1116 R 0.964*  1117 D 0.998**  1118 H 0.998**  1119 I 0.962*  1121 E 0.968*  1122 F 0.968*  1123 T 0.999**  1124 P 0.999**  1125 L 1.000**  1126 A 1.000**  1127 C 0.999**  1128 L 0.968*  1129 K 0.999**  1130 S 0.969*  1131 P 0.998**  1132 R 0.998**  1133 Y 0.998**  1136 N 0.999**  1137 D 0.967*  1139 S 0.969*  1140 N 0.969*  1142 P 1.000**  1143 A 0.973*  1144 R 0.999**  1145 R 0.971*  1146 S 1.000**  1147 H 0.999**  1148 S 0.999**  1149 R 1.000**  1150 S 0.999**  1151 K 0.999**  1152 K 0.977*  1153 T 0.999**  1154 M 0.971*  1155 F 0.994**  1156 T 0.968*  1157 H 0.968*  1158 W 1.000**  1159 R 0.974*  1160 G 1.000**  1161 I 0.972*  1163 Y 0.999**  1164 S 0.998**  1165 V 0.970*  1166 L 0.979*  1168 S 1.000**  1169 V 0.998**  1172 Y 0.998**  1173 S 0.999**  1174 N 0.999**  1175 K 1.000**  1176 H 0.999**  1178 R 0.997**  1179 S 0.964*  1180 S 0.970*  1181 A 0.969*  1183 Y 1.000**  1184 R 1.000**  1185 Y 0.998**  1186 S 1.000**  1188 S 1.000**  1189 G 0.980*  1191 E 0.974*  1192 T 0.968*  1193 S 1.000**  1194 V 1.000** |
|  |  | Ma0 | 8192.972018 |  |  |  |  | ω0=0.00000 ω1=1.00000 ω2=1.00000 |  |
| *PLXDC2* | *Lagopus muta* | Ma | 4193.329567 | Ma vs Ma0 | 232.48773 | 1 | 1.70932E-52 | ω0=0.04193 ω1=1.00000 ω2=999.00000 | 1 M 1.000**  2 A 0.999**  3 R 0.997**  4 L 0.952*  5 R 0.992**  6 R 0.997**  7 S 0.997**  8 K 0.968*  9 L 0.996**  11 A 0.964*  12 G 0.999**  13 F 0.997**  14 L 0.955*  15 L 0.999**  17 F 1.000**  18 Q 0.996**  19 F 0.961*  20 L 0.990**  21 S 0.970*  22 E 0.997**  24 C 0.981*  25 Q 0.998**  26 L 0.958*  27 A 0.997**  28 G 0.997**  29 G 0.997**  30 E 1.000**  31 T 0.997**  32 A 0.994**  33 S 0.994**  413 T 0.842 |
|  |  | Ma0 | 4309.573432 |  |  |  |  | ω0=0.03872  ω1=1.00000 ω2=1.00000 |  |
| *PLXDC2* | *Meleagris gallopavo* | Ma | 4237.879856 | Ma vs Ma0 | 108.38521 | 1 | 2.21303E-25 | ω0=0.00000 ω1=1.00000 ω2=656.91608 | 64 W 0.994**  65 R 0.996**  66 R 0.934  67 H 0.997**  68 S 0.999**  69 E 0.958*  70 S 0.999**  71 L 0.954*  72 K 0.944  74 V 0.997**  75 N 0.978*  76 T 0.946  78 R 0.941  79 A 0.961*  80 S 0.997**  81 M 0.996**  82 G 0.996**  83 Q 0.999**  84 D 0.962*  85 S 0.995**  86 S 0.940  110 T 0.952*  111 D 0.944  112 H 0.948  113 N 0.997**  115 Y 0.996**  116 T 0.994**  117 S 0.992**  253 R 0.999**  254 I 0.992**  255 I 0.994**  257 G 0.993**  258 Y 0.993**  259 K 0.916  260 E 0.999**  332 S 0.942 |
|  |  | Ma0 | 4292.072461 |  |  |  |  | ω0=0.00000 ω1=1.00000 ω2=1.00000 |  |
| *PLXDC2* | *Gallus gallus* | Ma | 4182.710812 | Ma vs Ma0 | 261.457224 | 1 | 8.25809E-59 | ω0=0.00000 ω1=1.00000 ω2=471.54400 | 42 V 0.975*  51 E 0.871  157 R 0.998**  158 V 1.000**  159 N 0.969*  160 L 0.999**  162 F 0.972*  163 D 1.000**  284 V 0.980*  285 V 0.999**  286 V 0.980*  288 R 1.000**  289 I 0.997**  291 Q 1.000**  292 I 1.000**  294 N 0.985*  295 V 0.999**  296 R 0.997**  305 R 0.977*  306 V 0.979*  307 E 0.976*  308 L 0.999**  311 S 1.000**  312 K 0.999**  313 I 1.000**  314 T 1.000**  315 N 0.999**  316 L 0.999**  317 S 0.999**  318 A 0.982*  319 V 0.999**  320 E 0.978*  321 M 0.999**  322 I 0.999**  324 L 0.999**  354 C 1.000**  355 S 0.979*  356 S 0.999**  376 D 0.980*  377 K 0.981*  378 I 0.833  379 C 0.999**  380 E 0.999**  381 K 0.976*  382 N 0.989*  384 D 0.999**  385 T 0.986*  405 V 0.979*  406 L 0.983*  408 T 0.981*  409 T 0.999**  433 L 0.975*  434 K 1.000**  436 N 0.979*  438 A 0.979*  439 S 0.999**  489 F 0.999**  490 I 0.983*  491 E 0.983*  493 R 0.991**  494 P 0.976*  495 S 0.999**  496 R 0.987*  497 W 1.000**  498 P 0.998**  501 K 0.999** |
|  |  | Ma0 | 4313.439424 |  |  |  |  | ω0=0.02206 ω1=1.00000 ω2=1.00000 |  |
| *TUBGCP3* | *Gallus gallus* | Ma | 5438.076238 | Ma vs Ma0 | 2249.273628 | 1 | 0 | ω0=0.02133 ω1=1.00000 ω2=228.78985 | 5 D 0.973*  7 K 0.981*  8 S 0.986*  10 N 0.999**  15 N 0.975*  23 K 0.977*  24 S 0.999**  25 E 0.995** |
|  |  | Ma0 | 5474.367875 |  |  |  |  | ω0=0.01912 ω1=1.00000 ω2=1.00000 |  |

**Table S6c** The results of branch-site model for Sphenisciformes.

| **Gene** | **The branch of** | **Model** | **-lnL** | **Model**  **comparison** | **2ΔlnL** | **df** | ***P*-value** | **Parameters** | **Positive site**  **(PP≥80%)** |
| --- | --- | --- | --- | --- | --- | --- | --- | --- | --- |
| *GHSR* | *Pygoscelis adeliae* | Ma | 2812.557027 | Ma vs Ma0 | 13.311396 | 1 | 0.000263798 | ω0=0.00545  ω1=1.00000  ω2=976.28458 | 1 M 0.933  3 E 0.970* |
|  |  | Ma0 | 2819.212725 |  |  |  |  | ω0= 0.00538  ω1=1.00000  ω2=1.00000 |  |
| *GHSR* | *Aptenodytes patagonicus* | Ma | 2791.740016 | Ma vs Ma0 | 56.620306 | 1 | 5.28631E-14 | ω0=0.00533  ω1=1.00000  ω2=999.00000 | 2 R 0.819  5 S 0.989*  10 G 0.998**  13 N 0.987*  14 R 0.821  15 T 0.955*  18 E 0.990*  19 P 0.985* |
|  |  | Ma0 | 2820.050169 |  |  |  |  | ω0=0.00542  ω1= 1.00000  ω2=1.00000 |  |
| *GRB10* | Pygoscelis papua | Ma | 3926.352000 | Ma vs Ma0 | 26.553326 | 1 | 2.56363E-07 | ω0 = 0.00000  ω1= 1.00000  ω2=999.00000 | 154 K 0.923  155 V 0.881  156 F 0.980*  158 E 0.992**  159 D 0.923  162 S 0.936  163 K 0.916  164 V 0.988*  168 L 0.978*  169 A 0.984*  170 D 0.905  171 M 0.876  176 L 0.867  177 C 0.952*  178 Q 0.964*  182 Y 0.987*  183 K 0.987*  185 H 0.940  186 C 0.985*  188 D 0.989*  189 D 0.982*  190 N 0.935  191 S 0.954*  192 W 0.926  203 L 0.935 |
|  |  | Ma0 | 3939.628663 |  |  |  |  | ω0=0.00000  ω1=1.000  ω2=1.000 |  |
| *GRB10* | *Aptenodytes patagonicus* | Ma | 3917.557294 | Ma vs Ma0 | 44.142738 | 1 | 3.05282E-11 | ω0=0.00000  ω1=1.000  ω2=999.00000 | 154 K 0.943  155 V 0.907  156 F 0.985*  158 E 0.994**  159 D 0.942  162 S 0.949  163 K 0.939  164 V 0.991**  165 V 0.969*  166 E 0.967*  168 L 0.983*  169 A 0.988*  170 D 0.927  171 M 0.906  176 L 0.892  177 C 0.960*  178 Q 0.836  182 Y 0.990*  183 K 0.991**  185 H 0.952*  186 C 0.988*  188 D 0.992**  189 D 0.986*  190 N 0.970*  191 S 0.961*  192 W 0.944  203 L 0.949 |
|  |  | Ma0 | 3939.628663 |  |  |  |  | ω0=0.00000  ω1=1.000  ω2=1.000 |  |
| *NCAPG* | *Aptenodytes forsteri* | Ma | 4998.787164 | Ma vs Ma0 | 10.975682 | 1 | 0.000923153 | ω0=0.21574  ω1 =1.000  ω2=999.00000 | 212 C 0.841 |
|  |  | Ma0 | 5004.275005 |  |  |  |  | ω0=0.10121  ω1=1.000  ω2=1.000 |  |
| *OBSL1* | *Pygoscelis adeliae* | Ma | 12512.986404 | Ma vs Ma0 | 204.790702 | 1 | 1.88128E-46 | ω0=0.02276  ω1 =1.000  ω2=999.00000 | 1020 A 0.991**  1089 L 0.802  1096 I 0.802  1101 D 0.972*  1105 S 0.820  1107 H 0.995**  1109 S 0.913  1389 P 0.862  1391 V 0.993**  1393 E 0.999**  1395 I 0.802  1396 T 0.889  1398 L 0.847  1402 T 0.986*  1407 E 0.815  1408 D 0.990*  1409 A 0.810  1411 F 0.855  1412 K 0.992**  1413 C 0.994**  1414 L 0.889  1415 V 0.850  1419 D 0.998**  1423 T 0.999**  1426 L 0.994**  1430 P 0.888  1431 V 0.876  1432 V 0.995**  1440 T 0.996**  1442 S 0.863  1444 L 0.817  1445 C 0.996**  1450 L 0.873  1451 R 0.906  1453 C 0.998**  1454 Q 0.870  1458 A 0.990*  1550 Y 0.861  1551 R 0.889  1553 E 0.868  1554 S 0.994**  1555 L 0.994**  1556 H 0.884  1558 R 0.998**  1559 T 0.985*  1560 Q 0.869  1561 A 0.992**  1562 R 0.860  1564 C 0.986*  1566 E 0.869  1567 P 0.862  1716 Q 0.887  1721 E 0.894  1723 A 0.995**  1731 D 0.998**  1732 A 0.837  1734 D 0.999** |
|  |  | Ma0 | 12615.381755 |  |  |  |  | ω0=0.02136  ω1=1.000  ω2=1.000 |  |
| *OBSL1* | *Aptenodytes forsteri* | Ma | 12372.77101 | Ma vs Ma0 | 490.358154 | 1 | 1.1909E-108 | ω0=0.04310  ω1=1.000  ω2=999.00000 | 720 A 0.981*  721 P 0.871  722 Q 0.629  724 R 0.830  726 A 0.816  727 P 0.998**  728 P 0.835  729 K 0.873  730 A 0.965*  732 R 0.997**  733 L 0.833  734 R 0.970*  735 E 0.979*  736 V 0.999**  740 L 0.894  741 P 0.888  743 L 0.995**  745 E 0.988*  748 V 0.852  750 P 0.887  751 L 0.838  758 L 0.998**  762 K 0.804  763 A 0.981*  765 P 0.999**  766 L 0.876  767 D 0.869  768 D 0.831  769 I 0.886  770 I 0.986*  771 A 0.854  772 V 0.852  773 Q 0.873  774 A 0.993**  777 C 0.998**  778 V 0.948  779 R 0.975*  782 L 0.906  784 R 0.889  785 S 0.986*  787 G 0.899  789 S 0.992**  793 T 0.991**  794 Y 0.865  795 T 0.985*  800 D 0.986*  802 A 0.862  804 S 0.994**  805 F 0.884  806 V 0.990*  810 T 0.870  902 E 0.980*  903 A 0.882  904 P 0.890  906 R 0.999**  908 I 0.992**  909 S 0.999**  910 S 0.997**  911 N 0.993**  913 D 0.949  914 A 0.999**  915 P 0.912  917 H 0.873  918 A 0.988*  919 Y 1.000**  920 T 0.993**  924 R 0.914  926 E 0.995**  928 W 0.996**  933 R 0.917  934 L 0.840  935 A 0.989*  939 R 0.871  941 Y 0.989*  943 V 0.921  945 E 0.859  946 G 0.959*  950 G 0.995**  952 C 0.968*  954 V 0.864  955 L 0.983*  956 E 0.999**  957 Q 0.992**  960 P 0.999**  961 R 0.995**  962 H 0.873  965 V 0.999**  966 L 0.983*  968 C 0.996**  970 R 0.997**  972 Q 0.999**  974 A 0.897  976 E 0.996**  977 F 0.899  978 V 0.993**  983 G 0.981*  985 S 0.898  986 V 0.864  987 F 0.998**  988 Y 0.899  989 T 0.976*  1924 S 0.998** |
|  |  | Ma0 | 12617.95009 |  |  |  |  | ω0=0.03962  ω1=1.000  ω2=1.000 |  |
| *ATP11A* | *Aptenodytes forsteri* | Ma | 5484.388877 | Ma vs Ma0 | 8.077114 | 1 | 0.004482778 | ω0=0.00000 ω1=1.00000 ω2=354.45687 | 794 K 0.880 |
|  |  | Ma0 | 5488.427434 |  |  |  |  | ω0=0.00000 ω1=1.00000 ω2=1.00000 |  |
| *ATP11A* | *Spheniscus magellanicus* | Ma | 5478.468324 | Ma vs Ma0 | 12.413204 | 1 | 0.000426309 | ω0=0.00000 ω1=1.00000 ω2=999.00000 | 39 S 0.884  40 S 0.976*  41 K 0.886  482 N 0.884 |
|  |  | Ma0 | 5484.674926 |  |  |  |  | ω0=0.00000 ω1=1.00000 ω2=1.00000 |  |
