# Supplementary material for: Insight Into Body Size Evolution in Aves: Based on Some Body Size‐Related Genes
Source: Integr Zool. 2024 Dec 11;20(6):1124–35. doi: 10.1111/1749-4877.12927 (PMC12618961; doi:10.1111/1749-4877.12927)
Supplement: Supplementary file 8 — Table S7 The annotation information of functional sites and domains of branch‐site model analysis for each dataset (identity with sites of human). a is for 56 avian species; b is for Galliformes; c is for Sphenisciformes [file INZ2-20-1124-s011.docx]

**Table S7a** The annotation information of functional sites and domains of branch-site model analysis for 56 avian species (identity with sites of human).

| **Proteins** | **branch** | **sites** | **Site feature** **(UniPprot or InterPro)** |
| --- | --- | --- | --- |
| EIF2AK3 | *Cuculus canorus* | 661I (678E) | Topological domain-Cytoplasmic |
| OBSL1 | *Dromaius novaehollandiae* | 922 G (915) | Disulfide bond |
| OBSL1 | *Struthio camelus* | 1122 D (1117) | Disulfide bond |
| PLAG1 | *Hirundo rustica* | 72 A | Zinc finger-C2H2-type zinc fingers 3 interacts with DNA-binding site G-clusterinc fingers. |
| IGF2BP1 | *Pygoscelis adeliae* | 441 A (442) | Close to Phosphothreonine(446) |
| IGF2BP1 | *Struthio camelus* | 78 N (78) | Close to Phosphothreonine(73) |
| IGF2BP1 | *Struthio camelus* | 440 V (441)  442 F (443)  443 S (444)  445 W (446) | KH3 and KH4 mediate association with the cytoskeleton (441,443,444)  Phosphothreonine(446) |
| IGF2BP1 | *Parus major* | 445 A (446T) | Phosphothreonine(446) |
| ATP11A | *Callipepla squamata* | 265 V  266 A  267 I  268 Y | Topological domain-Cytoplasmic |
| ATP11A | *Corvus kubaryi* | 815 K | Topological domain-Cytoplasmic |
| ATP11A | *Aptenodytes forsteri* | 807 I (804) | Binding site-ATP |
| ATP11A | *Patagioenas fasciata* | 1123 C  1155 N  1156 S  1159 K  1160 K  1186 R  1189 A | Topological domain-Cytoplasmic |
| PLXDC2 | *Aptenodytes patagonicus* | 491 E 0.893 | Topological domain-Cytoplasmic |
| PLXDC2 | *Oxyura jamaicensis* | 159 N (160) | Glycosylation |

Numbers in parentheses are indicated as functional sites adjacent to those detected sites.

**Table S7b** The annotation information of functional sites and domains of branch-site model analysis for Galliformes (identity with sites of human).

| **Proteins** | **branch** | **sites** | **Site feature (UniPprot or InterPro)** |
| --- | --- | --- | --- |
| GALNS | *Colinus virginianus* | 10 W (10)  22 N (32)  27 L (36) | PhobiusSIGNAL_PEPTIDE, Phobius SIGNAL_PEPTIDE_H_REGION  Sulfatase enzyme, Phobius SIGNAL_PEPTIDE,  Sulfatase enzyme, Alkaline-phosphatase-like, core domain superfamily, SignalP_Euk SignalP-noTM |
| GALNS | *Meleagris gallopavo* | 392 H (401)  430 P (439)  433 F (442) | Close to Glycosylation, (423) |
| PLXDC2 | *Gallus gallus* | 160 L (160) | Glycosylation (160) |
| TUBGCP3 | *Gallus gallus* | 5 D  7 K  10 N | Close to Modified residue (2) |

**Table S7c** The annotation information of functional sites and domains of branch-site model analysis for Sphenisciformes (identity with sites of human).

| **Proteins** | **branch** | **sites** | **Site feature (UniPprot or InterPro)** |
| --- | --- | --- | --- |
| GRB10 | *Pygoscelis papua* | 170 D (187)  171 M (188)  176 L (193)  177 C (194)  178 Q (195)  182 Y (199)  183 K (200) | Ras-associating domain; Ubiquitin-like domain superfamily (InterPro); Phosphatidylinositol 3-kinase Catalytic Subunit; Chain A, domain 1 (InterPro); Ras-associating (RA) domain found in growth factor receptor-bound (Grb) protein 10 (InterPro) |
| GRB10 | *Aptenodytes patagonicus* | 171 M (188)  176 L (193)  177 C (194)  178 Q (195)  182 Y (199)  183 K (200) | Ras-associating domain; Ubiquitin-like domain superfamily (InterPro); Phosphatidylinositol 3-kinase Catalytic Subunit; Chain A, domain 1 (InterPro); Ras-associating (RA) domain found in growth factor receptor-bound (Grb) protein 10 (InterPro) |
| ATP11A | *Aptenodytes forsteri* | 794 K (807) | Topological domain; Close to Binding site (804); P-type ATPase, haloacid dehalogenase domain (InterPro) |
| ATP11A | *Spheniscus magellanicus* | 39 S (52) | Topological domain; P-type ATPase, N-terminal (InterPro) |
