# Supplementary material for: Insight Into Body Size Evolution in Aves: Based on Some Body Size‐Related Genes
Source: Integr Zool. 2024 Dec 11;20(6):1124–35. doi: 10.1111/1749-4877.12927 (PMC12618961; doi:10.1111/1749-4877.12927)
Supplement: Supplementary file 9 — Table S8 The results of parallel/convergent amino acid substitution sites [file INZ2-20-1124-s004.docx]

**Table S8** The results of parallel/convergent amino acid substitution sites.

| Branches | Genes | Sites | AA change | Observed number | Expected number | P-value |
| --- | --- | --- | --- | --- | --- | --- |
| a vs c | *EIF2AK3*  *NCAPG* | 702  857  80  484 | R-G  T-A  Y-C  A-V | 4 | 0 | 0 |
| a vs d | *TUBGCP3*  *GHSR*  *IGFBP7*  *OBSL1*  *NCAPG*  *ACAN* | 694  342  35  1790  384  911  383  426  467 | M-I  S-L  P-A  P-L  S-T  S-T  P-S  G-S  T-M | 9 | 0 | 0 |
| a vs e | *GALNS*  *TNS3*  *PLOD1*  *OBSL1*  *NCAPG*  *ACAN* | 428  961  1558  567  103  134  681  685  688  694  1102  1329  484  694  694 | R-Q  R-C  V-I  T-I  Q-R  T-A  Q-R  T-M  S-L  A-V  G-E  I-V  A-V  T-M  V-I | 15 | 0 | 0 |
| a vs f | *TNS3*  *PLOD1*  *NCAPG*  *ACAN* | 994  1023  476  300  484  519  991  426  1917 | S-P  P-A  M-L  I-T  A-V  R-K  V-A  G-S  T-A | 9 | 0 | 0 |
| a vs g | *TNS3*  *OBSL1*  *NCAPG*  *ACAN*  *GALNS* | 1179  134  304  2143  70  426  1782  1842  438 | R-Q  T-A  I-V  G-S  N-D  G-S  T-A  A-T  A-V | 9 | 0 | 0 |
| a vs h | *GALNS*  *OBSL1*  *ACAN* | 428  1023  1029  1430  1436 | R-Q  A-T  R-A  I-S  V/I-A | 5 | 0 | 0 |
| a vs i | *PLOD1*  *ACAN* | 730  1430 | A-V  I-S | 2 | 0 | 0 |
| a vs j | *PLXDC2*  *OBSL1*  *NCAPG*  *ACAN* | 101  18  685  479  952  1351  1430 | D-E  T-M  T-M  D-E  E-D  I-V  I-S | 7 | 0 | 0 |
| a vs k | *TNS3*  *OBSL1*  *ACAN*  *IGF2BP1* | 895  304  1591  1790  2001  499  2075  12 | R-Q  I-V  G-S  P-L  R-Q  R-Q  I-T  N-S | 8 | 0 | 0 |
| a vs l | *TUBGCP3*  *OBSL1*  *NCAPG*  *ACAN* | 217  694  1329  928  426  734 | T-A  M-I  I-V  I-T  G-S  V-M | 6 | 0 | 0 |
| b vs c | *ACAN* | 1406 | F-S | 1 | 0 | 0 |
| c vs d | *EIF2AK3*  *NCAPG* | 850  315  434 | I-T  F-L  I-T | 3 | 0 | 0 |
| c vs e | *NCAPG* | 484  549  692 | A-V  M-T  T-S | 3 | 0 | 0 |
| c vs f | *NCAPG*  *ACAN* | 484  684  928  1371 | A-V  E-K  I-V  V-I | 4 | 0 | 0 |
| c vs g | *ACAN* | 1000  1679 | A-T  V-L | 2 | 0 | 0 |
| c vs i | *ACAN* | 328 | V/A-I | 1 | 0 | 0 |
| c vs j | *NCAPG* | 479 | D-E | 1 | 0 | 0 |
| d vs e | *TNS3*  *OBSL1*  *ACAN* | 795  297  694  1371  1389 | A-T  T-A  A-V  V-I  F-T | 5 |  |  |
| d vs f | *NCAPG*  *ACAN* | 717  426  679  1389  1391 | A-T  G-S  S-T  F-T  M-V | 5 | 0 | 0 |
| d vs g | *PLOD1*  *OBSL1*  *ACAN* | 730  211  258  409  426 | I-V  A-V  R-G  A-T  G-S | 5 | 0 | 0 |
| d vs h | *EIF2AK3* | 732 | H-N | 1 | 0 | 0 |
| d vs i | *OBSL1* | 1719  2071 | T-M  Q-R | 2 | 0 | 0 |
| d vs j | *OBSL1*  *NCAPG* | 288  28  240 | I-V  V-I  M-L | 3 | 0 | 0 |
| d vs k | *EIF2AK3*  *OBSL1* | 309  1790 | R-Q  P-L | 2 | 0 | 0 |
| d vs l | *TUBGCP3*  *PLOD1*  *OBSL1*  *NCAPG*  *ACAN* | 694  290  974  619  1377  1389 | M-I  I-V  T-A  E-D  H-Q  F-T | 6 | 0 | 0 |
| e vs f | *EIF2AK3*  *TNS3*  *NCAPG*  *ACAN* | 696  817  484  777  967  1371  1389  1397  1761  1807 | T-P  A-T  A-V  V-M  A-V  V-M  F-T  Q-H  T-K  A-M | 10 | 0 | 0 |
| e vs g | *OBSL1* | 2  134  1097 | E-D  T-A  P-L | 3 | 0 | 0 |
| e vs h | *GALNS*  *EIF2AK3*  *TNS3*  *NCAPG* | 428  768  817  58 | R-Q  C-R  A-T  I-V | 4 | 0 | 0 |
| e vs i | *IGFBP7* | 280 | E-P | 1 | 0 | 0 |
| e vs j | *OBSL1*  *NCAPG*  *ACAN* | 639  685  107  1823 | A-V  T-M  E-Q  H-Q | 4 | 0 | 0 |
| e vs k | *TUBGCP3*  *OBSL1* | 770  382  1949 | V-G  A-S  T-M | 3 | 0 | 0 |
| e vs l | *TUBGCP3*  *EIF2AK3*  *OBSL1*  *ACAN* | 769  806  685  1702  1537  1764 | R-G  H-Q  T-M  G-E  H/Q-R  I-V | 6 | 0 | 0 |
| f vs g | *ACAN* | 16  337  426 | A-T  D-H  G-S | 3 | 0 | 0 |
| f vs h | *TNS3* | 817 | A-T | 1 | 0 | 0 |
| f vs i | *NCAPG* | 444 | S-P | 1 | 0 | 0 |
| f vs j | *NCAPG* | 878 | N/D-E | 1 | 0 | 0 |
| f vs k | *ACAN* | 1439 | V-I | 1 | 0 | 0 |
| f vs l | *TNS3*  *NCAPG*  *ACAN* | 696  362  915  426  1389  1431 | T-A  M-I  E-Q  G-S  F-T  I-T | 6 | 0 | 0 |
| g vs k | *OBSL1*  *NCAPG* | 79  304  49 | V-I  I-V  I-V | 3 | 0 | 0 |
| g vs l | *TNS3*  *PLOD1*  *OBSL1*  *ACAN* | 735  102  1329  426 | G-S  I-V  I-V  G-S | 4 | 0 | 0 |
| g vs i | *TNS3*  *OBSL1* | 69  264 | R-L  A-V | 2 | 0 | 0 |
| h vs i | *PLXDC2*  *OBSL1*  *NCAPG* | 378  56  417  676 | I-V  M-V  T-S  T-N | 4 | 0 | 0 |
| h vs j | *IGFBP7*  *OBSL1*  *EIF2AK3* | 15  848  550 | L-M  R-H  P-S | 3 | 0 | 0 |
| h vs k | *OBSL1* | 923 | Q-E | 1 | 0 | 0 |
| h vs l | *OBSL1*  *NCAPG*  *ACAN* | 1065  683  1411 | I-V  G/T-S  I-T | 3 | 0 | 0 |
| i vs j | *PLOD1* | 730 | V-M | 1 | 0 | 0 |
| i vs k | *OBSL1*  *ACAN* | 1392  1439 | D-N  V-I | 2 | 0 | 0 |
| i vs l | *IGF2BP1* | 46  55 | 1. S   E-I | 2 | 0 | 0 |
| k vs l | *TUBGCP3*  *OBSL1*  *ACAN* | 770  153  2097  2127  637  1380 | V-G  K-E  D-N  T-M  Q-E  H-Q | 6 | 0 | 0 |
| j vs k | *TNS3* | 1224 | Q-E | 1 | 0 | 0 |
| j vs l | *ACAN* | 1433 | T-I | 1 | 0 | 0 |
| J vs kl | *PLXDC2* | 103 | T-A | 1 | 0 | 0 |
