# Supplementary material for: Insight Into Body Size Evolution in Aves: Based on Some Body Size‐Related Genes
Source: Integr Zool. 2024 Dec 11;20(6):1124–35. doi: 10.1111/1749-4877.12927 (PMC12618961; doi:10.1111/1749-4877.12927)
Supplement: Supplementary file 10 — Table S9 The annotation information of functional sites and domains of parallel/convergent analysis (identity with sites of human) [file INZ2-20-1124-s003.docx]

**Table S9** Convergent/parallel functional site analysis (identity with sites of human)

| **Proteins** | **sites** | **Site feature (UniProt or** **InterPro)** |
| --- | --- | --- |
| *OBSL1* | 18  79  56  848  382  304  297  153  923  258  288  264 | Interaction with TTN  Disulfide bond  Disulfide bond  Disulfide bond  Disulfide bond  Disulfide bond  Disulfide bond  Disulfide bond  Disulfide bond  Immunoglobulin domain subtype (InterPro)  Immunoglobulin domain subtype (InterPro)  Immunoglobulin domain subtype (InterPro) |
| *NCAPG* | 928  952  240  107  300 | Modified residue (929)  Modified residue (959)  Armadillo-type fold (InterPro)  Armadillo-type fold (InterPro)  Armadillo-type fold (InterPro) |
| *GSHR* | 342 | Topological domain |
| *EIF2AK3* | 550  702  806 | Topological domain; Modified residue (555)  Topological domain  Topological domain |
| *TNS3* | 735  696 | Modified residue(735)  Modified residue(698, 692) |
| *ACAN* | 383  734  328  1537  337  637 | Close to Glycosylation, (387)  Close to Glycosylation, (738)  Close to Glycosylation, (333)  Close to Glycosylation, (1530)  Disulfide bond, ( 273-348)  Disulfide bond, (622-643) |
| *IGF2BP1* | 12 | Modified residue, |
| *PLXDC2* | 101  103  378 | Close to Glycosylation, (103)  Glycosylation  Close to Glycosylation, (103) |
| *TUBGCP3* | 217 | Polar residues (210-230) |
| *IGFBP7* | 35 | Disulfide bond, (32-57) |

Numbers in parentheses are indicated as functional sites adjacent to those detected sites.
