# Supplementary material for: Insight Into Body Size Evolution in Aves: Based on Some Body Size‐Related Genes
Source: Integr Zool. 2024 Dec 11;20(6):1124–35. doi: 10.1111/1749-4877.12927 (PMC12618961; doi:10.1111/1749-4877.12927)
Supplement: Supplementary file 11 — Table S10 Phylogenetic generalized least squares (PGLS) regression analyses of gene evolutionary rates and body mass [file INZ2-20-1124-s010.docx]

**Table S10** Phylogenetic generalized least squares (PGLS) regression analyses of gene evolutionary rates and body mass.

| **Gene** | R**^2^** | ***F*** | ***P*** | ***λ*** |
| --- | --- | --- | --- | --- |
| *IGF2BP1* | 0.138 | 7.715 | 0.0014 | 1.000 |
